# Systematic Review on Irrational Use of Medicines in China and Vietnam

**DOI:** 10.1371/journal.pone.0117710

**Published:** 2015-03-20

**Authors:** Wenhui Mao, Huyen Vu, Zening Xie, Wen Chen, Shenglan Tang

**Affiliations:** 1 School of Public Health, Fudan University, P.O. Box 187, 138 Yi Xue Yuan Road, 200032, Shanghai, China; 2 Collaborative Innovation Center of Social Risks Governance in Health; 3 Duke Global Health Institute, Duke University, 310 Trent Drive, 27708, Durham, NC, USA; 4 Duke Kunshan University, No. 8 Duke Avenue, 215316, Kunshan, Jiangsu Province, China; California Pacific Medicial Center Research Institute, UNITED STATES

## Abstract

**Background:**

Irrational use of medicines has been an issue concerned all over the world and the outlooks in developing countries are more severe. This study aimed to assess the different patterns of irrational use of medicines and its influential factors in China and Vietnam.

**Methods:**

A systematic review was performed on both published and grey literatures in English, Chinese and Vietnamese languages between 1993 and 2013 based on the WHO framework. Quality assessment was conducted on the basis of the Critical Appraisal Skills Programme. Key indicators were analyzed to compare the irrational use of medicines in two countries.

**Results:**

A total of 67 published works about China and 29 about Vietnam were included, the majority of which were cross-sectional prescription studies in both China and Vietnam. Irrational use of medicines was found in both the countries but issues with polypharmacy as well as overuse of antibiotics were more severe in Vietnam while overuse of injections was unique to China. Various patterns of irrational use were also indicated between urban and rural areas, and among different levels of hospitals. Rarely does literature focus on the analysis of influential factors of irrational use of medicines. While lack of proper knowledge from both providers and patients were the most recognized influential factors in both countries, economic incentives from pharmaceutical companies in China, and weak control and regulation over prescriptions in Vietnam were the main factors attributed to this issue.

**Conclusion:**

Severe irrational use of medicines has been abundantly evidenced in both China and Vietnam, highlighting the importance of policy interventions on the issue. However, limited evidence on the appropriateness or its compliance (conformity) to guidelines of prescription has been found. In addition, convincing evidence on the underlying explanation of this issue is lacking, although economic incentives, health insurance coverage, and knowledge of service providers and users have been implied to be factors influencing irrational drug use.

## Introduction

It is estimated that 60% of medicines in public health facilities and 70% of medicines in private facilities were prescribed and sold inappropriately in developing countries, which leads to the decrease in safety and quality of health care as well as enormous wastage of health resources [1].Statistics suggest irrational use of medicines was listed among the top 10 causes of morbidity and mortality in the U.S. [2,3] and it cost approximately US$870 million to provide care and treatment for those who were admitted to the hospital due to adverse medical events in the UK [4,5].

The most common problems associated with irrational use of medicines include selection of medicines without consideration for cost-effectiveness and efficacy, inefficient procurement of unnecessarily expensive drugs, failure to prescribe medicines in accordance with standard treatment protocols, poor dispensing practices resulting in medication errors, improper patients adherence to dosing schedules and treatment regimens, and inappropriate self-medication [6–9]. In developing countries less than half of all acute viral upper respiratory tract infection and viral diarrhea cases were treated with antibiotics correctly [8,10–13]. In addition, more than 50% of patients worldwide failed to take their medications properly [8]. Either overuse or underuse of antibiotics can also result in serious antimicrobial resistance [14].

Despite expanding medicine expenditure in both China and Vietnam, irrational use of medicines has been a severe problem in both of the countries. It is estimated that about half of antibiotic prescriptions in China were medically unnecessary [15–17]. In Vietnam, 35%–60% of medicines prescribed in rural clinics were antibiotics [18–20]. Another study also indicated that 80% of the antibiotics were purchased from private pharmacies without a prescription [21]. Health systems in China and Vietnam share some commonalities but also some distinctions. A comparison of irrational use of medicines between the two countries could exchange lessons learnt for both of the countries, which would have to be useful to both of the countries and other developing countries with similar conditions.

Specifically, the health care system in general and pharmaceuticals in particular in China and Vietnam are similar in the way that both nations adopted market mechanisms in their healthcare and pharmaceutical sectors [22].The commercialization of public health sector in both China and Vietnam has encouraged the development and availability of medicines and other health products, but also stimulated over-prescription of medicines. Although the pharmaceutical sectors in both nations have focused their development on both modern and traditional medicines to support the affordability and availability of medicines and have contributed to transforming quality of health over the past decades, the majority of patients still have to pay high out-of-pocket costs of medicine [23]. In addition, many patients still have stronger preference for imported drugs to domestically-manufactured/ generic drugs.

In the meantime, China and Vietnam have their distinct characteristics in health systems and different implementation plans of healthcare reform. Since the 1950s, the public sector has been the dominant power of health care services in China. Government financial support had been the main source of compensation for public hospitals since hospital services were provided at their prime cost. Although government health expenditure grew 30 times its initial amount from 1980 to 2005, its proportion out of the total health expenditure declined from 36.2% to 17.9% [24]. Profits from sale of medicines, whose expenditure accounted for over 40% of the total health expenditure, replaced government investment and became the major income source for hospitals. Under the government policy, hospitals were officially permitted to put mark-up rate of 25% and 20–25%, respectively for western medicines and for manufactured Traditional Chinese Medicines. As a result, hospitals and doctors tended to prescribe expensive drugs and/or more drugs than necessary to maximize their profits [25,26]. However, since drug price was always under strict governmental scrutiny and regulation, and that patients were more sensitive to expensive drugs, the quantity of medicines per prescription became the means to increase profits [27–29] (Please note: Total revenue of medicines = (Retail price—Prime price) * Quantity; Since the retail price has a ceiling set by government and prime price was determined by manufactures, it was easier and unnoticeable for physicians increase the quantity of medicine than necessary). Health insurance schemes, which covered over 95% of the population by 2012, played an increasingly important role in improving the access of health care [30]. Despite the high population coverage, health insurance schemes have not fully executed their role originally bestowed as a third payer and have instead limited influence on health service delivery, especially when retrospective payment methods such as fee-for-service were widely applied as the main provider payment method. Herein, the lack of negotiation power from service users or health insurance in improving quality and quantity of health services mechanism, might also contribute to the overuse of medicines [31].

As Vietnam reformed its health system in the late 1980s by introducing market-oriented mechanisms, public hospitals were allowed to operate private pharmacies. Private pharmacies have become the dominant medicine supplier in the market, representing 60% of the market share on pharmaceutical sales [22,32–34]. However, the transition from a centralized pharmaceutical supply system to a commercialized mechanism has caused some negative impacts on Vietnam’s health care system. Easy accessibility to pharmacies has led to the overuse of prescribed drugs, especially antibiotics. Although access to essential medicines and vaccines has positively changed and improved the health status of the population, Vietnam’s Ministry of Health reported that for the majority of the population, “access to the right medicines at the time they need them remains a major challenge” due to high drug prices, poor quality of medicines and vaccines, irrational selection and use of drugs, unsustainable drug manufacture systems, and a lack of a financial support system for drug procurement [35].Statistics in 2008 indicated a 40% of the country’s total health care expenditure accounted for by the cost of medicine, of which up to 72% out of total were household out-of-pocket share [36]. Noticeably, self-medication makes up 40% of total household out-of-pocket spending [37].

Both Chinese and Vietnamese governments have taken actions to address the problems in the past decades. China launched a new health system reform in 2009 with the objective of equitable access to basic health care. Recently policies developed, such as the National Essential Medicines and Zero Mark-up for Drug Sale, are intended to improve the access, affordability, as well as the rational use of medicine. However, the original goals of the policies have not been achieved yet. By the same token, Vietnam is facing similar challenges in controlling irrational use of medicines. Despite the challenges in irrational use of antibiotics and other prescribed medicines, there is a lack of effective control and regulation mechanisms for drug use and for prescribing, especially at district and commune levels [19]. The Vietnam National Drug Policy of 1996 highlighted rational antibiotic use and set rules for drug prescriptions and therapeutic treatments in clinical settings. Vietnam’s Ministry of Health has defined antibiotics as a prescription-only drug since 2003 [38,39]; however, there have been no regulations to penalize non-compliance.

The purpose of this research is to assess the landscape of irrational use of medicines in each of the two countries. Specifically, it aims to (1) describe the situation of irrational use of medicines in each country and (2) examine and analyze influential factors.

## Methods

### Scope of our study

**Fig 1:**
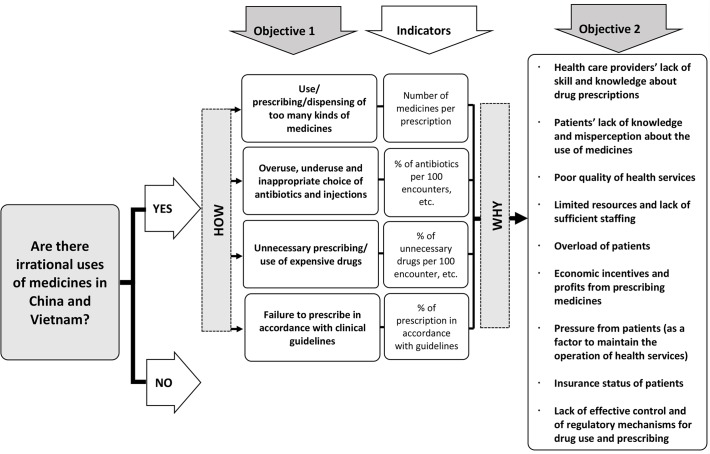
Conceptual framework for the rational use of medicines.

Irrational use of medicines has broad definition which may vary from place to place or from disease to disease. In order to facilitate further comparability with other studies and to make this review more structured, a conceptual framework was developed, according to the WHO’s Access to Medicine framework [6](Fig. 1) in which the definition of irrational use of medicines has been divided into four detailed patterns and the influential factors have been summarized into nine categories. The data synthesis was also conducted, using this framework.

### Search strategy

To identify eligible studies on irrational use of medicines from 1993–2013 in Chinese, English and Vietnamese, major databases such as PubMed, EMBASE, PQDT, WOK, CBM, CNKI (China National Knowledge Infrastructure), Wangfang and Chongqing VIP database, were searched. A pilot search in PubMed had been conducted to ensure the comprehensiveness of key search terms. We used a mixture of free text and index terms to maximize retrieval of potentially relevant studies. Our PubMed key word search included (medicine OR drug OR pharmaceutical product OR antibiotic OR injection) AND (rational OR ration OR proper OR over OR abuse OR misuse) AND (use OR prescribe OR prescription OR dispense OR self-medication OR self-treatment OR self-prescript OR health-seeking behavior OR polypharmacy OR over-the-counter) AND (China OR Chinese OR Vietnam).

Since a database on Vietnamese literature was not available, a manual search was conducted (the search list can be found in S3 Table). Grey literature, technical reports, policy implementation reports, white paper, etc. were searched from Google Scholar and related government websites, or accessed via professional contacts with other researchers and colleagues. The reference lists of identified articles were also reviewed.

### Study selection

Eligibility of literature was identified independently by three reviewers (WM, HV and ZX) strictly based on the inclusion and exclusion criteria in Table 1. First, the title and abstract were screened and the full-text followed. Excluded articles were recorded with an explanation for exclusion. Any inconsistencies among the reviewers were settled by discussion and resolved with final consensus. The process was managed using EndNote software.

**Table 1:** Inclusion and exclusion criteria.

***Inclusion Criteria***	
1. *Types of the literature*:	peer-reviewed studies, PhD dissertations, official laws and regulations, policy documents and executive documents
2. *Publication language*:	English, Chinese and Vietnamese
3. *Study Design*:	Objective 1: At least one of the following study designs should be used: case-control study, cohort study, retrospective study, cross-sectional study, before and after comparison, pilot study, and stimulated methods.
	Objective 2: At least one of the following study designs should be used: case studies, qualitative studies, case-control study, cohort study, retrospective study, cross-sectional study, before and after comparison, pilot study
4. *Data collection*:	Objective 1: At least one of the methods should be used: household survey, population-based survey (patient, service user), facility survey (service providers, pharmacies, or health facilities), or prescription analysis
	Objective 2: At least one of the methods should be used: interview, household survey, population-based survey (patient, service user), facility survey (service providers, pharmacies, or health facilities), or prescription analysis
5. *Study population*:	General population of all ages, or patients of both common or specific diseases (i.e., cold, cough, reproductive transmitted infection, TB, HIV/AIDS
***Exclusion Criteria***	
1. *Publication type*	Commentary, editorial, letter to the editor, books and book chapter, lecture, systematic review, narrative review and meta-analysis review
2. *Study design*:	Biopharmaceutical, laboratory studies and studies that did not report study designs/data collection methods/sampling framework/sample size/study population.
3. *Sample size*:	Objective 1: Studies with a sample size of less than 50 people and/or 30 prescriptions per facility and/or minimum of 2 pharmacies/health facilities
	Objective 2: Except for case studies, studies with a sample size less than 50 people and/or 30 prescriptions per facility and/or minimum of 2 pharmacies/health facilities
4. Studies that are about:	Traditional medicine or drug/addictive substance abuse (i.e., heroin, etc.); about Hong Kong, Macao and Taiwan
5. *Duplications*:	If two or more studies share similar databases, study population and/or study topic, the quality of the studies should be assessed by two reviewers. The study with the best study design should be included, and others should be excluded. If these studies also have similar study designs, the most recently published study should be included

**Fig 2:**
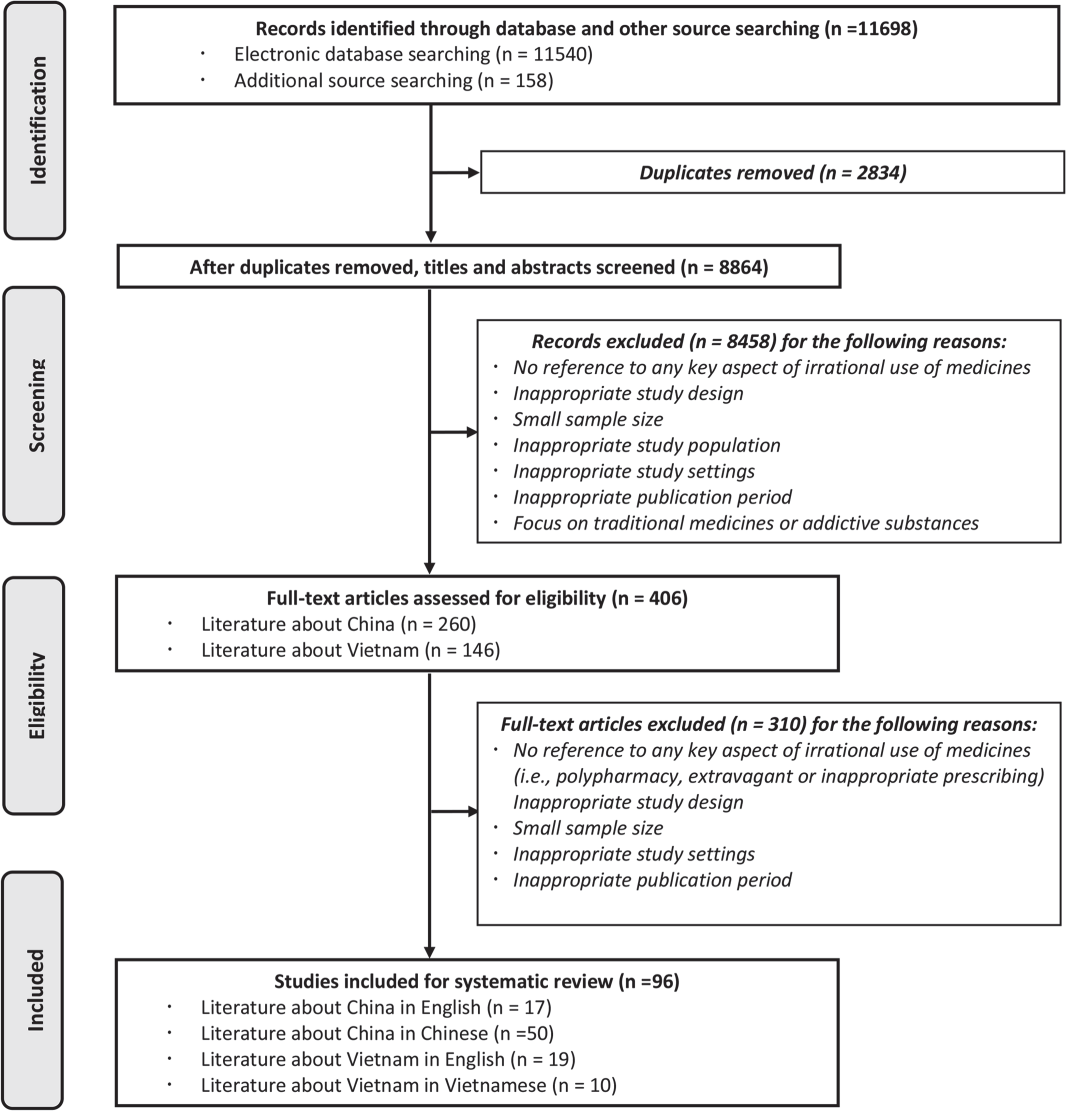
PRISMA 2009 Flow Diagram [40].

Fig. 2 presents an illustration of the search output. The initial search yielded 11698 potentially relevant articles and narrowed down to 96 studies to be finally included, among which 67 were about China and 29 were about Vietnam.

### Quality assessment

Since studies of different designs were included in this review, the checklists from the Critical Appraisal Skills Programme (CASP) were adapted and developed to assess the quality of the studies. More specifically, the CASP checklists for qualitative research, case control and cohort studies were developed into a quality assessment rating system [41]. With 10 questions for each list, 10 points were distributed to each question.

Three reviewers (WM, HV and ZX) rated the literature separately and the average score was calculated as the final score for the literature. Disagreement among raters was addressed through discussion until consensus on all scores was met.

### Data synthesis

The conceptual framework (Fig. 1) was also employed to instruct data synthesis [6]. Patterns of irrational use of medicines and its influential factors were summarized. Quantitative indicators were mainly used to assess the irrational use of medicines (objective 1) while qualitative information was used to assess the influential factors (objective 2). Both types of data were abstracted independently by three reviewers (WM, HV and ZX) in accordance with the framework. More specifically, key indicators derived from the WHO manual [42] were extracted in their original form without any extra calculations or alternations from the reviewers. The indicators collected were number of medicines per prescription, percentage of antibiotics and injections prescribed per 100 encounters, number of antibiotics per encounter, number of injections per encounter and percentage of broad spectrum medications prescribed per 100 encounters, etc. Any inconsistencies were discussed and finalized by the review team members.

## Results

### Description of studies

Table 2 and S1 Table present all the characteristics for eligible studies. Among the eligible literature, the average score of the quality assessment was 7.67 for China and 7.86 for Vietnam. About two-thirds of the articles were published in the past 5 years. Most included articles were peer-reviewed. In China most of included studies were carried out in the urban areas, while more Vietnamese studies used study sites from the rural areas. There was far more literature assessing the irrational use of medicines (objective 1) than studies examining or analyzing factors contributing to it (objective 2).The majority of the literature was cross-sectional research. Drug prescription surveys were conducted by most studies in China; other data collection methods were also used at different frequencies in China and Vietnam. Patients selected from clinical settings were the key study population in both countries, followed by the general population. Clinicians and pharmacy staff received more attention in Vietnam. Since China and Vietnam are burdened with different spectrums of diseases, more studies focused on children in Vietnam, particularly in regards to diarrhea management, pneumonia susceptibility and antibiotic overuse. While 9.1% of the population in China was the elderly over the age of 65 in 2012 [43], only one study analyzed the elderly as the target population. More studies in China focused on primary care facilities, while the pharmacies were more studied in Vietnam.

**Table 2:** Characteristics of included studies.

Characteristics	China (N = 67)	Vietnam (N = 29)
**Quality assessment(0–10)**	**7.67**	**7.86**
***Published year***		
1993–2002	6(9.0%)	6(20.7%)
2003–2008	15(22.4%)	5(17.2%)
2009–2013	46(68.6%)	18(62.1%)
***Research Type***		
Peer reviewed article	54(80.6%)	27(93.2%)
Degree dissertation	13(19.4%)	1(3.4%)
Report	-	1(3.4%)
***Geographical area***		
Urban	28(41.8%)	8(27.6%)
Rural	19(28.2%)	15(51.7%)
Both urban and rural	20(30.0%)	6(20.7%)
***Study objective(Multiple*, *if applicable)***		
Assess the irrational use of medicines	65(97.0%)	24(82.8%)
Describe/analyze factors contributing to the irrational use of medicines	38(56.7%)	17(58.6%)
***Study design(Multiple*, *if applicable)***		
Cross-sectional/Case-Control	47(70.1%)	19(66.5%)
Time series/Surveillance/Cohort	20(29.9%)	2(6.9%)
Qualitative study/case study	4(6.0%)	5(17.2%)
Simulated client method	1(1.5%)	3(10.38%)
***Data collection method(Multiple*, *if applicable)***		
Population-based survey	16(23.9%)	12(29.4%)
Facility survey	11(16.4%)	5(17.2%)
Prescription survey	42(62.7%)	4(13.8%)
Medical record review	9(13.4%)	1(3.4%)
Pharmacy survey	4(6.0%)	7(24.1%)
Qualitative interview (i.e., in-depth, focus group)	6(9.0%)	10(34.5%)
Bio-test	-	5(17.2%)
***Study population(Multiple*, *if applicable)***		
Clinicians	6(9.0%)	13(44.8%)
Pharmacy staff	1(1.5%)	9(31.0%)
Patient	52(77.6%)	18(62.1%)
General population	10(14.9%)	18(62.1%)
Adults	6(9.0%)	1(3.4%)
Elderly	1(1.5%)	-
Children	6(9.0%)	11(37.9%)
***Facility types (Multiple*, *if applicable)***		
Tertiary or Secondary Hospital(for CN)/Hospital(for VN)	22(32.8%)	-
Primary healthcare facility(for CN)/Outpatient clinic(for VN)	44(65.7%)	3(10.3%)
Pharmacy	4(6.0%)	7(24.1%)

### Findings on patterns of irrational use of medicines (Objective 1)

Findings presented in Table 3 were listed, in accordance with the framework (Fig. 1). No eligible studies were found to assess whether or not unnecessary or expensive drugs were prescribed, and whether or not the prescription was in accordance with clinical guidelines.

**Table 3:** Pattern of irrational use of medicines by indicators in China and Vietnam.

Summary by measuring indicators	China	Vietnam
***Number of medicines per prescription***	**n = 30**	**n = 8**
0.01–2.00 drugs	1(3.3%)	2(25.0%)
2.01–3.00 drugs	15(50.0%)	-
3.01–4.00 drugs	12(40.0%)	4(50.0%)
4.01 and more drugs	2(6.7%)	2(25.0%)
***Percentage of polypharmacy***	**n = 7**	**n = 0**
0%–10%	2(28.5%)	-
11%–20%	2(28.5%)	-
21%–30%	3(43.0%)	-
***Percentage of antibiotics prescribed per 100 encounters***	**n = 41**	**n = 13**
0%–20%	-	1(7.7%)
21%–40%	6(14.6%)	1(7.7%)
41%–60%	24(58.5%)	3(23.2%)
61%–80%	10(24.4%)	4(30.7%)
81%–100%	1(2.5%)	4(30.7%)
***Number of antibiotics per encounter***	**n = 7**	**n = 3**
0.51–1.00 drugs	6(85.7%)	-
1.01–1.50 drugs	1(14.3%)	3(100%)
***Percentage of injections prescribed per 100 encounters***	**n = 33**	**n = 2**
0%–20%	4(12.1%)	1(50.0%)
21%–40%	12(37.4%)	1(50.0%)
41%–60%	16(48.5%)	-
61%–80%	1(3.0%)	-
***Percentage of self-medication with antibiotics***	**n = 6**	**n = 1**
0%–30%	2(33.3%)	-
31%–60%	3(50.0%)	-
61%–100%	1(16.7%)	1(100%)
***Percentage of antibiotics usage with wrong course***	**n = 3**	**n = 7**
0%–50%	-	3(42.9%)
51%–100%	3(100%)	4(57.1%)
% **of unnecessary drugs per 100 encounter**	**n = 0**	**n = 0**
% ***of prescription in accordance with guidelines***	**n = 0**	**n = 0**

Polypharmacy is a severe issue in both China and Vietnam. Vietnam’s problem with polypharmacy is suggested to be more challenging, indicated by the number of medicines per prescription [42].The majority of studies indicated there were 2–4 types of drug per prescriptions on average in China and 3–4 kinds in Vietnam. The median of the indicator is 2.94 in China and 3.75 in Vietnam (detailed information in Table 4 and S2 Table). Moreover, the median percentage of polypharmacy from seven articles evaluating polypharmacy in China was 15.91%. A trend was also found in China that this polypharmacy indicator had a higher median among studies conducted in rural as opposed to urban areas (3.13 vs.2.55). Among different levels of health facilities, the median of the tertiary and secondary hospitals was 2.37 while it was 3.07 for primary healthcare facilities.

**Table 4:** Median for the key indicators.

Distribution of key indicators	Median
***Number of medicines per prescription***	
China	2.94
Vietnam	3.75
***Percentage of antibiotics prescribed per 100 encounters***	
China	52.60
Vietnam	69.00
***Number of antibiotics per encounter***	
China	0.75
Vietnam	1.30
***Percentage of injections prescribed per 100 encounters***	
China	40.75
Vietnam	-

Antibiotics and injections are two important, but commonly overused and costly forms of drug therapy [42]. Antibiotics were irrationally prescribed in both countries, and more irrational use of antibiotics was found in Vietnam compared to China. The majority of literature from China reported that the percentage of antibiotics prescribed per 100 encounters was between 41–60%. One article about primary health facilities in rural China even suggested the percentage to be 87.56%. The median of this indicator among the studies was 52.60% with rural areas exhibiting a higher level at 55.14% compared to that of urban (48.58%). This indicator has a median of 69% in Vietnam and 4 out of 13 articles suggested over 80% of prescriptions were dispensed with antibiotics. The combination use of antibiotics further illustrated the issue with abuse in Vietnam. Compared to 0.80 in China, the median number of combination antibiotics per encounter was 1.30 in Vietnam (According to the U.S. Centers for Disease Control and Prevention, percentage of antibiotics prescribed per 100 encounters for person aged ≤ 14 years who had visited physician offices was 22.9% in 2007–2008, which was much more lower than most of the numbers reported by research in China and Vietnam.

Overuse of injections was only reported in China. Most studies indicated the percentage of injections prescribed per 100 encounters was between 21% and 60%. The median was 40.75%. Moreover, the median of rural areas was 43.00% while it was a bit lower in urban areas at 34.55%. The median was 34.6% for studies focused on tertiary and secondary hospitals—lower than that for primary healthcare facilities (40.88%).

The median percentage of self-medication with antibiotics was 47.4% in China, indicating the high prevalence of self-medicated antibiotics. No eligible study reported this indicator in Vietnam, but patients in both the countries tend to take antibiotics incorrectly.

### Findings on factors associated with irrational medicine use (Objective 2)

Although only a few studies focused on assessing and analyzing influential factors of irrational use of medicines, many studies described or derived potential influential factors from their assessment on irrational use. As a results, limited evidence is available to assess the extent to which each factor or attributes to life year loss, or resources waste. However, nine influential factors summarized in the framework were mentioned in articles with different frequency, and similarities and differences of them can be found between China and Vietnam, which may be used as a suggestion for further analysis on influential factors of irrational use of medicines (Table 5).

**Table 5:** Summary of influential factors of the irrational use of medicines in China and Vietnam.

Summary by influential factors	China (N = 67)	Vietnam (N = 29)
Health care providers’ lack of skills and knowledge	9(13.4%)	8(27.6%)
Patients’ lack of knowledge	14(20.9%)	10(33.5%)
Poor quality of health services	2(3.0%)	2(6.9%)
Health facility’s inadequate human resources and lack of qualified medical staff	–	3(10.3%)
Pressure from heavy patient load	2(3.0%)	–
Pressure from patients’ demand	1(1.5%)	–
Economic incentive and profits from prescribing medicines	20(30.0%)	4(13.8%)
Insurance status of patients	4(6.0%)	1(3.4%)
Lack of effective control and regulatory mechanisms on medicine use	3(4.5%)	7(24.1%)

In China, economic incentive from prescribing medicines has been regarded as a factor influencing irrational drug use by 30% of studies. A survey on the health providers found that over 70% of the providers regarded bonuses as an incentive given to doctors to prescribe more or expensive services [31]. Another study indicated that essential drugs were perceived as unprofitable for its low price and strict regulation policy [32]. Some study on economic incentive in Vietnam reported that private providers tended to pursue more profits by over-prescribing [34].

Studies in China and Vietnam shared the same view that the lack of knowledge, especially from the patients’ side, was one of the important influential factors for irrational use. In Vietnam, over four-fifths of service providers have a misperception that antibiotics were indicated for treatment of mild acute respiratory infections with fever [44]. A study on the overuse of injections in China indicated that many people believed that invasive procedures were more effective than taking oral medicines [45].

Lack of effective control and regulatory mechanisms for medicine use was mentioned by seven studies in Vietnam as an important factor of irrational use [34,46]. One study raised concerns over the low compliance with prescription regulation that almost every pharmacy dispensed corticosteroids without prescriptions [47]. However, the evidence was not obvious in China.

In addition, pressure from heavy workloads and patient demands, and the insurance status of patients were factors concerned in China. Furthermore, issues related to inadequate human resources in health facilities and the lack of qualified medical staff were more prominent in Vietnam.

## Discussion

### Current practice of irrational use of medicines and its influential factors

A great challenge for the rational use of medicines in both countries is the management of excessive kinds of medicines and the abuse of antibiotics. The situation was more severe in Vietnam than in China. More specifically, people in Vietnam tended to use more kinds of medicines than the Chinese, and the percentage of overuse or misuse of antibiotics was higher. In China, however, polypharmacy and irrational use of antibiotics were found to be more severe in the rural areas than in the urban area, and in primary healthcare facilities than in tertiary or secondary hospitals. We cannot draw any conclusion for Vietnam on this issue, as the number of studies on this perspective is limited.

China, compared with Vietnam, had better population coverage of health insurance and stricter regulations on price, prescription and licensing for health providers. However, the factors of the policy on mark-up rate allowing service providers to make profits, “fee-for-service” payment methods, and the non-cost based prices for health services have been repetitively suggested in many studies as the direct link of perverse economic incentives to over-prescription [31,46,48]. Additionally, many people of the general population faithfully embraced that the effectiveness of antibiotics and injections over other regimens, and that these therapies tend to be actively demanded when visiting doctors. Since patients in China have the freedom to choose whom to seek medical attention from as health providers, some doctors admittedly responded that they would (have) prescribe antibiotics and use injections upon patient requests in order to please their clients[31,49].

In the meantime, people in rural areas were less educated, generally speaking, than their counterparts in urban areas; thus, people with a relatively better knowledge base about medications were less likely to be living in rural settings. Furthermore, insurance service benefit packages in rural China were not as comprehensive as those in the urban area. The management of pharmacies and the surveillance of the quality of medicines were also weaker in rural areas. All these factors contributed to the vulnerability of rural populations to irrational use of medicines, so that polypharmacy and abuse of antibiotics were much more severe in rural areas, which was in accordance with our findings from key indicators. Another trend we found in China was that the issue of irrational use of medicines was worse in primary healthcare facilities than in tertiary or secondary hospitals. A reasonable explanation could be that primary healthcare facilities, which are relatively small in scale, rely more on the revenues from drug sale. The improved access to primary healthcare facilities, which introduced more competition among primary healthcare facilities, could also be a contributing factor. That is to say, it is much easier for patients to replace one primary healthcare facility with another health facility so that doctors in primary healthcare facilities are keener to satisfy patients by prescribing antibiotics or injections. However, further research on the comparison of irrational use of medicines between urban and rural area or primary and tertiary/ secondary hospitals is necessary since our deduction was based on limited number of research and may not be comprehensive.

Vietnam, on the other hand, introduced its health system reform after its economic reform in 1986. Although the public health sector remains to play its important role for inpatient services, privatized pharmacy operated by hospital dominated the retail market of medicines. Meanwhile, Vietnam’s public health insurance scheme is yet to standardize as less than half of its people are covered. This might be one important reason why a high proportion of self-medication among residents was found in Vietnam [21,34]. Patients’ lack of proper medical knowledge, especially in rural areas [50], could be easily affected or even led astray by pharmacists or market promotion activities of the private sector. As a result, some patients were unfortunately administered incorrect medications [51]. Much worse, the weak implementation of the regulations in the pharmaceutical market and under-qualified staff led to more irrational use of medicines [34].

### Good practice and lessons learnt for further improvement on irrational use of medicines

Although neither China nor Vietnam has been able to prevent irrational use of medicines, lessons and good practice can be learnt from each other, and for other developing countries with similar problems. As one of the most powerful tools recommended by WHO to improve the rational use of medicine [52], the Essential Drug List (EDL) was introduced in Vietnam about two decades ago, and the Law on Pharmacy was then issued in 2005 [53]. But the implementation of regulations was relatively weak and the EDL was out-of-date, which made it difficult to achieve the ultimate goal of Vietnam’s health policy. China introduced the National Essential Medicine Policy (EMP) in 2009, along with the centralized procurement of essential medicines and implementation of the Zero Mark-up Policy. After two years of implementation, the overall performances of rational use of medicines and cost control were still not significantly improved [54]. Lessons can be learnt from Vietnam that the implementation of policy is as important as the policy itself.

Perverse economic incentives have negative effects on the rational use of medicine. For China, policies to remove mark-up, such as the Zero Mark-up Policy, and reform of income distribution systems, should be considered. The rational prescribing should be considered as one of the indicators for the assessment of physicians’ performance. Price control on retail pharmacies, regulation of marketing campaigns as well as prescription management in private pharmacies are recommended to be the priority in Vietnam [55], so as to limit and regulate profit seeking behavior of the private pharmacies.

Health insurance is another key factor in improving the rational use of medicine. As a third payer, especially in China where wide population coverage has expanded its influence on hospitals, health insurance agencies should uphold accountability of quality control. Quality-oriented and prepayment mechanisms, among which rational use of medicines can be an important indicator, could build a new connection between payment and quality service outputs, which could promote the rational use of medicine as well.

### Limitations

This review did not find enough evidence, especially quantitative evidence, to form a comparable analysis of the influential factors of irrational use. The same reason also made it impractical to assess whether unnecessary or expensive drugs were prescribed or whether the prescription was prescribed or taken in accordance with clinical guidelines. The clinical needs of patients are fundamental to the rational use of medicine; nonetheless, the cost to patients’ communities should be considered with the same level of criticality and urgency as the personal burden of disease. Thus, more research should be conducted.

Another limitation might have been sample sizes, as the sample sizes of the studies reviewed herein were not balanced between the two countries, which may have introduced certain types of bias. The homogeneity of included studies was not as uniform as had been expected. In addition, convincing evidence on the explanation of this issue is absent, which adds difficulty for formulating evidence-based policy recommendation.

## Final Remarks

Severe irrational use of medicines has been abundantly evidence in both China and Vietnam, thus highlighting the importance of policy interventions required to tackle this issue. Based on the evidence from available studies, polypharmacy, overuse and abuse of antibiotics should be the focus of the interventions. Some but not sufficient evidences clued that in China, irrational use of medicines was more concerning in rural area than urban area, and in primary healthcare facility than in secondary or tertiary hospital. However, further research should be taken to find more evidence for this preliminary deduction. Although strong evidence on the influential factors and their influential mechanism of irrational use is absent, lack of knowledge from patient and provider was recognized by many articles, thus highlights the importance of education on providers and patients in both countries. Economic incentives, health insurance status and the enforcement of policy should be regarded as potential influential factors and further research on their impact on irrational use should be conducted.

## Supporting Information

S1 Checklist PRISMA 2009 checklist. (DOC)

S1 Table Detailed Characteristics of included studies. (DOCX)

S2 Table Sample size and key indicators of included studies. (DOCX)

S3 Table Manual searched list. (DOCX)
