# Supplementary material for: Systematic Review on Irrational Use of Medicines in China and Vietnam
Source: PLoS One. 2015 Mar 20;10(3):e0117710. doi: 10.1371/journal.pone.0117710 (PMC4368648; doi:10.1371/journal.pone.0117710)
Supplement: S1 Table — (DOCX) [file pone.0117710.s002.docx]

**Table S1 Detailed Characteristics of included studies**

| # | **Country** | **Authors** | **Title** | **Quality Assessment Rating(0-10)** | **Published Year** | **Research Type** | **Geographical Area** | **Study Area(Provinces)** | **Study Objectives** | **Study Design** | **Data Collection** | **Study Population** | **Age Group** | **Facility Type** |
| --- | --- | --- | --- | --- | --- | --- | --- | --- | --- | --- | --- | --- | --- | --- |
| 1 | CN | Bai H | Investigation of store state of family medicine-chest and drug taking safety | 8 | 2008 | PhD Dissertation | Urban | Hunan | Assess the Irrational Use and Describe the Influential Factors | Cross-sectional | Population Based Survey | General Population |  |  |
| 2 | CN | Bi P | Family self-medication and antibiotics abuse for children and juveniles in a Chinese city | 8 | 2000 | Peer Reviewed Article | Urban | Hefei | Assess the Irrational Use and Describe the Influential Factors | Cross-sectional | Population Based Survey | General Population | Children |  |
| 3 | CN | Cai W | Analysis of antibiotics uses in Shanghai during the first half of 2005 | 8 | 2006 | Peer Reviewed Article | Urban | Shanghai | Assess the Irrational Use | Cross-sectional | Facility Survey; Prescription Survey | Patient |  | Tertiary/Secondary |
| 4 | VN | Chalker J | STD management by private pharmacies in Hanoi: practice and knowledge of drug sellers | 7 | 2000 | Peer Reviewed Article | Urban | Hanoi | Assess the Irrational Use and Describe the Influential Factors | Simulated client method | Population Based Survey; Bio-test | Patient |  |  |
| 5 | CN | Chang X | Research on the condition and influential factors of the prescribing in Beijing community health service centers | 7 | 2012 | PhD Dissertation | Urban | Beijing | Assess the Irrational Use and Describe the Influential Factors | Qualitative | Facility Survey; Prescription Survey | Patient |  | Primary |
| 6 | CN | Chen F | Retrospective analysis of compatible infusion prescriptions for outpatients and emergency patients of 11 hospitals in Tianjin |  | 2010 | Peer Reviewed Article | Urban | Tianjin | Assess the Irrational Use | Cross-sectional | Prescription Survey | Patient |  | Tertiary/Secondary |
| 7 | CN | Chen J | International indicators for rational drug use in outpatient prescriptions of 21 medical institutions in Shanghai Songjiang District | 8 | 2009 | Peer Reviewed Article | Urban | Shanghai | Assess the Irrational Use | Cross-sectional | Prescription Survey | Patient |  | Tertiary/Secondary; Primary |
| 8 | CN | Chen W | Availability and use of essential medicines in China: manufacturing, supply, and prescribing in Shandong and Gansu provinces | 8 | 2010 | Peer Reviewed Article | Urban & Rural | Shandong and Gansu Province | Assess the Irrational Use and Describe the Influential Factors | Cross-sectional | Prescription Survey; Pharmacy Survey; Interview | Pharmacy Staff; Patient |  | Primary; Pharmacy |
| 9 | VN | Chuc NT | Management of childhood acute respiratory infections at private pharmacies in Vietnam | 7 | 2001 | Peer Reviewed Article | Urban | Hanoi | Assess the Irrational Use and Describe the Influential Factors | Simulated Client method | Pharmacy Survey |  |  | Pharmacy |
| 10 | VN | Chuc NTK | “Doi moi” and private pharmacies: a case study on dispensing and financial issues in Hanoi, Vietnam | 7 | 1999 | Peer Reviewed Article | Urban | Hanoi | Assess the Irrational Use and Describe the Influential Factors | Case study | Pharmacy Survey; Interview |  |  | Pharmacy |
| 11 | CN | Dong H | Health financing policies: Providers' opinions and prescribing behavior in rural China | 8 | 1999 | Peer Reviewed Article | Rural | Jiangsu, Anhui, and Jiangxi | Describe the Influential Factors | Cross-sectional and Qualitative | Population Based Survey | Clinician |  | Tertiary/Secondary; Primary |
| 12 | CN | Dong L | Polypharmacy and its correlates in village health clinics across 10 provinces of Western China | 8 | 2010 | Peer Reviewed Article | Rural | Xinjiang, Neimenggu, Qinghai, Gansu, Ningxia, Sichuan, Chongqing, Guizhou, Jiangxi, Guangxi | Assess the Irrational Use and Describe the Influential Factors | Cross-sectional | Population Based Survey; Prescription Survey; Interview | Clinician; Patient |  | Primary |
| 13 | CN | Dong L | Antibiotic prescribing patterns in village health clinics across 10 provinces of Western China | 8 | 2008 | Peer Reviewed Article | Rural | Xinjiang, Neimenggu, Qinghai, Gansu, Ningxia, Sichuan, Chongqing, Guizhou, Jiangxi, Guangxi | Assess the Irrational Use and Describe the Influential Factors | Cross-sectional | Prescription Survey | Patient |  | Primary |
| 14 | CN | Dong L | Comprehensive evaluation of prescription indicators for rational drug use of village doctors in 40 counties in west China | 8 | 2012 | Peer Reviewed Article | Rural | 10 provinces of western China | Assess the Irrational Use | Cross-sectional | Prescription Survey | Patient |  | Primary |
| 15 | VN | Duong DV | Availability of antibiotics as over-the-counter drugs in pharmacies: a threat to public health in Vietnam | 9 | 2011 | Peer Reviewed Article | Urban | Hanoi | Assess the Irrational Use and Describe the Influential Factors | Cross-sectional | Pharmacy Survey; Interview | Pharmacy Staff; Patient | Children | Pharmacy |
| 16 | CN | Han S | Can Price controls reduce pharmaceutical expenses? A case study of antibacterial expenditures in 12 Chinese hospitals from 1996 to 2005 | 6 | 2013 | Peer Reviewed Article | Urban | Beijing | Describe the Influential Factors | Time Series or Surveillance | Facility Survey |  |  | Tertiary/Secondary |
| 17 | CN | He Z | Evaluation of prescriptions in 12 secondary hospitals in Nantong area between June and Dec. in 2007 | 8 | 2008 | Peer Reviewed Article | Urban & Rural | Jiangsu | Assess the Irrational Use | Cross-sectional | Prescription Survey | Patient |  | Tertiary/Secondary |
| 18 | VN | Hoa NQ | Antibiotics and paediatric acute respiratory infections in rural Vietnam: health-care providers' knowledge, practical competence and reported practice | 8 | 2009 | Peer Reviewed Article | Rural | Ba Vi District | Assess the Irrational Use and Describe the Influential Factors | Cross-sectional | Population Based Survey | Clinician |  |  |
| 19 | VN | Hoa NQ | Decreased Streptococcus pneumoniae susceptibiity to oral antibiotics among children in rural Vietnam: a community study | 8 | 2010 | Peer Reviewed Article | Rural | Ba Vi District | Assess the Irrational Use | Cross-sectional | Population Based Survey; Bio-test | General Population | Children |  |
| 20 | VN | Hoa NQ | Drug use and health-seeking behavior for childhood illness in Vietnam-A qualitative study | 7 | 2007 | Peer Reviewed Article | Urban & Rural | Hanoi and Ha Tay Province | Assess the Irrational Use and Describe the Influential Factors | Qualitative (Focus group) | Interview | Patient | Children |  |
| 21 | VN | Hoan LT | Drug use among children under 5 with respiratory illness and/or diarrhoea in a rural district of Vietnam | 8 | 2009 | Peer Reviewed Article | Rural | Ba Vi District | Assess the Irrational Use and Describe the Influential Factors | Cross-sectional | Population Based Survey; Interview | Patient | Children |  |
| 22 | VN | Hoang MH | Hoạt động cung ứng thuốc tại bệnh viện Hữu Nghị – Thực trạng và một số giải pháp | 7 | 2012 | PhD Dissertation | Urban | Hanoi | Assess the Irrational Use | Case Study | Facility Survey |  |  | Primary |
| 23 | VN | Hoang TT | Thực trạng sử dụng thuốc cho nhóm bệnh nhân bảo hiểm y tế tại trạm y tế xã | 7 | 2012 | Grey Literature | Rural | Ha Nam, Khanh Hoa, Kien Giang & Gia Lai | Assess the Irrational Use | Cross-sectional | Medical record Review | Patient |  | Tertiary/Secondary |
| 24 | CN | Jiang M | Status quo on prescription antibiotics in retail pharmacies of Shaanxi Province | 8 | 2013 | Peer Reviewed Article | Urban | Shaanxi | Assess the Irrational Use and Describe the Influential Factors | Cross-sectional and Simulated Client | Pharmacy Survey |  | Adult; Children | Pharmacy |
| 25 | CN | Jin C | Framing a global health risk from the bottom-up: User perceptions and practices around antibiotics in four villages in China | 7 | 2011 | Peer Reviewed Article | Rural | Hubei and Shandong | Assess the Irrational Use and Describe the Influential Factors | Cross-sectional and Qualitative | Interview | General Population |  |  |
| 26 | VN | Kaljee LM | Rural and urban Vietnamese mothers utilization of healthcare resources for children under 6 years with pneumonia and associated symptoms | 7 | 2010 | Peer Reviewed Article | Urban & Rural | Nha Trang City & Ninh Hoa District | Assess the Irrational Use and Describe the Influential Factors | Cross-sectional | Population Based Survey | General Population | Children |  |
| 27 | VN | Larsson M | Antibiotic medication and bacterial resistance to antibiotics: a survey of children in a Vietnamese community | 9 | 2000 | Peer Reviewed Article | Rural | Ba Vi District | Assess the Irrational Use and Describe the Influential Factors | Cohort | Population Based Survey | General Population | Children |  |
| 28 | VN | Larsson M | Private pharmacy staff in Hanoi dispensing steroids - theory and practice | 8 | 2006 | Peer Reviewed Article | Urban | Hanoi | Assess the Irrational Use and Describe the Influential Factors | Simulated client method | Pharmacy Survey; Interview | Pharmacy Staff |  | Pharmacy |
| 29 | VN | Larsson M | Overprescribing of antibiotics to children in rural Vietnam |  | 2005 | Peer Reviewed Article | Rural | Ba Vi District | Assess the Irrational Use | Cohort | Population Based Survey; Bio-test | Patient | Children |  |
| 30 | VN | Le TH | Drug use and self-medication among children with respiratory illness or diarrhea in a rural district in Vietnam: a qualitative study | 9 | 2011 | Peer Reviewed Article | Rural | Ba Vi District | Assess the Irrational Use and Describe the Influential Factors | Qualitative (in-depth interviews & focus group) | Pharmacy Survey; Interview | Pharmacy Staff; Patient | Children | Pharmacy |
| 31 | CN | Li F | Availability of essential medicines research of community health services in Guangzhou | 6 | 2011 | PhD Dissertation | Urban | Guangdong Province | Assess the Irrational Use and Describe the Influential Factors | Cross-sectional | Prescription Survey | Patient |  | Primary |
| 32 | CN | Li F | Investigation of utilization situation of antibiotics among residents in Beijing | 8 | 2008 | Peer Reviewed Article | Urban | Beijing | Assess the Irrational Use and Describe the Influential Factors | Cross-sectional | Population Based Survey | General Population |  |  |
| 33 | CN | Li J | Analysis on 82241 clinical prescriptions of 21 hospitals in Beijing | 6 | 2003 | Peer Reviewed Article | Urban | Beijing | Assess the Irrational Use | Cross-sectional | Prescription Survey | Patient | Elderly | Tertiary/Secondary; Primary |
| 34 | CN | Li L | Investigation of current situation of rational drug use among residents in three cities, Yunnan Province | 8 | 2012 | Peer Reviewed Article | Urban & Rural | Yunnan | Assess the Irrational Use and Describe the Influential Factors | Cross-sectional | Population Based Survey | General Population | Adult |  |
| 35 | CN | Li P | Study on strategies of improving access to essential medicines in rural of China | 6 | 2009 | PhD Dissertation | Rural | Hubei Province | Assess the Irrational Use | Cross-sectional | Prescription Survey | Patient |  | Tertiary/Secondary; Primary |
| 36 | CN | Li Y | Overprescribing In China, driven by financial incentives, results in very high use of antibiotics, injections, and corticosteroids | 9 | 2012 | Peer Reviewed Article | Urban | country wide | Assess the Irrational Use and Describe the Influential Factors | Time Series or Surveillance | Prescription Survey | Patient |  | Primary |
| 37 | CN | Li Y | The status and effect study of the policy implementation of essential drug in Chinese city community health institutions | 8 | 2011 | PhD Dissertation | Urban | country wide | Assess the Irrational Use and Describe the Influential Factors | Time Series or Surveillance | Prescription Survey | Patient |  | Primary |
| 38 | CN | Liang X | Unnecessary use of antibiotics for inpatient children with pneumonia in two counties of rural China | 8 | 2011 | Peer Reviewed Article | Rural | Hubei and Sichuan provinces | Assess the Irrational Use and Describe the Influential Factors | Cross-sectional | Facility Survey; Medical record Review; Interview | Patient |  | Tertiary/Secondary; Primary |
| 39 | CN | Lin L | Evaluation of the implementation of essential medicines system in Fujian primary care health institutions | 6 | 2012 | PhD Dissertation | Urban | Fujian Province | Assess the Irrational Use | Cross-sectional | Prescription Survey | Patient |  | Primary |
| 40 | CN | Ling N | Analysis on current situation of intravenous infusion in the community health service stations of Zhongshan City | 8 | 2010 | Peer Reviewed Article | Urban | Guangdong | Assess the Irrational Use and Describe the Influential Factors | Cross-sectional | Prescription Survey | Patient |  | Primary |
| 41 | CN | Liu J | Epidemiology study of prescription of antibacterial drugs of medical insurance inpatients in Changsha | 7 | 2011 | PhD Dissertation | Urban | Hunan | Assess the Irrational Use | Time Series or Surveillance | Medical record Review | Patient |  |  |
| 42 | CN | Lu l | Analysis of rational use of drug in community health service facilities | 9 | 2009 | Peer Reviewed Article | Urban | National Wide | Assess the Irrational Use and Describe the Influential Factors | Cross-sectional | Prescription Survey | Patient |  | Primary |
| 43 | CN | Luo H | Evaluation and analysis of outpatients and emergency prescriptions in 10 hospitals of Luzhou area and control measures | 8 | 2012 | Peer Reviewed Article | Urban & Rural | Sichuan | Assess the Irrational Use | Cross-sectional | Prescription Survey | Patient |  | Tertiary/Secondary |
| 44 | CN | Luo J | Study on the status and influential factors of self-medication with antibacterial agents among customers in pharmacies | 10 | 2009 | PhD Dissertation | Urban | Hunan | Assess the Irrational Use and Describe the Influential Factors | Cross-sectional | Pharmacy Survey |  |  | Pharmacy |
| 45 | CN | Luo J | Medicine use and cost analysis by prescriptions of community health service institutions in Chengdu and Shenyang | 8 | 2007 | Peer Reviewed Article | Urban | Sichuan and Liaoning | Assess the Irrational Use and Describe the Influential Factors | Cross-sectional | Prescription Survey | Patient |  | Primary |
| 46 | CN | Ma J | Medical costs and impacting factors on rural clinics of western rural areas in China | 6 | 2002 | Peer Reviewed Article | Rural | 40 county in Western China | Assess the Irrational Use and Describe the Influential Factors | Time Series or Surveillance | Facility Survey; Prescription Survey | Patient | Children | Primary |
| 47 | CN | Mo R | Investigation and countermeasures study on irrational antibiotics use in the community health centers | 8 | 2011 | Peer Reviewed Article | Urban | Guangdong | Assess the Irrational Use and Describe the Influential Factors | Cross-sectional | Population Based Survey; Prescription Survey | Clinician; Patient |  | Primary |
| 48 | VN | My HN | Reproductive Tract Infections in Northern Vietnam: Health Providers' Diagnostic Dilemmas | 8 | 2009 | Peer Reviewed Article | Urban | Hai Phong City | Assess the Irrational Use and Describe the Influential Factors | Cross-sectional + qualitative (mixed method) | Population Based Survey; Interview | Clinician; Patient | Children |  |
| 49 | VN | Nguyen TGH | Nghiên cứu tính bất hợp lý trong chỉ định thuốc và đề xuất nâng cao tính hợp lý trong sử dụng thuốc tại một số bệnh viện tại miền Bắc, Việt Nam | 7 | 2010 | Grey Literature | Urban & Rural | Cities and provinces in Northern Vietnam | Assess the Irrational Use and Describe the Influential Factors | Cross-sectional | Medical record Review; Interview | Patient; Pharmacy Staff |  | Primary; Tertiary/Secondary |
| 50 | VN | Nguyen TLH | Nhiễm ký sinh trùng sốt rét và thực trạng sử dụng thuốc sốt rét tự điều tri của người dân ngủ rẫy tại Vĩnh Thạnh, tỉnh Bình Định | 6 | 2010 | Peer Reviewed Article | Rural | Binh Dinh Province | Assess the Irrational Use and Describe the Influential Factors | Cross-sectional | Population Based Survey; Interview | General Population |  |  |
| 51 | VN | Nguyen H | The principal-agent problems in health care: evidence from prescribing patterns of private providers in Vietnam | 8 | 2011 | Peer Reviewed Article | Urban & Rural | Nation-wide | Assess the Irrational Use and Describe the Influential Factors | Cross-sectional | Population Based Survey | General Population |  |  |
| 52 | CN | O'Connor S | Physician control of pediatric antimicrobial use in Beijing, China, and its rural environs | 8 | 2001 | Peer Reviewed Article | Urban & Rural | Beijing and Hebei | Assess the Irrational Use | Cross-sectional and Qualitative | Pharmacy Survey; Interview | General Population | Children | Tertiary/Secondary; Primary; Pharmacy |
| 53 | VN | Okumura J | Drug utilisation and self-medication in rural communities in Vietnam | 9 | 2002 | Peer Reviewed Article | Rural | Nghe An, Thanh Hoa, Dong Thap, Can Tho Province | Assess the Irrational Use and Describe the Influential Factors | Cross-sectional | Population Based Survey; Interview | General Population | Adult |  |
| 54 | CN | Pan H | Prior knowledge, older age, and higher allowance are risk factors for self-medication with antibiotics among university students in Southern China | 8 | 2012 | Peer Reviewed Article | Urban | Guangdong | Assess the Irrational Use and Describe the Influential Factors | Cross-sectional | Population Based Survey | General Population | Adult |  |
| 55 | VN | Phuong HL | Acute undifferentiated fever in Binh Thuan province, Vietnam: imprecise clinical diagnosis and irrational pharmaco-therapy | 9 | 2006 | Peer Reviewed Article | Rural | Binh Thuan Province | Assess the Irrational Use | Cross-sectional | Population Based Survey; Bio-test | Patient |  |  |
| 56 | CN | Qin Y | The investigation of irrational use of medication in town hospitals and village health stations in Liaoning Province | 9 | 2006 | Peer Reviewed Article | Rural | Liaoning | Assess the Irrational Use and Describe the Influential Factors | Cross-sectional | Prescription Survey | Patient |  | Primary |
| 57 | CN | Qu J | An assessment on current situation of village clinics in Shandong Province--An assessment on standardization and quality of health services provided by village clinics | 7 | 2006 | Peer Reviewed Article | Rural | Shandong | Assess the Irrational Use | Cross-sectional | Population Based Survey; Facility Survey | Clinician | Adult; Children | Primary |
| 58 | VN | Quagliarello AB | Factors associated with carriage of penicillin-resistant Streptococcus pneumoniae among Vietnamese children: a rural-urban divide | 9 | 2003 | Peer Reviewed Article | Urban & Rural | Ho Chi Minh City & Dong Nai Province | Assess the Irrational Use and Describe the Influential Factors | Cross-sectional | Population Based Survey; Pharmacy Survey; Bio-test | General Population | Children | Pharmacy |
| 59 | CN | Ren Z | A survey of knowledge and behavior on antibiotic use among young people in China | 9 | 2012 | Peer Reviewed Article | Urban & Rural | Heilongjiang | Assess the Irrational Use and Describe the Influential Factors | Cross-sectional | Population Based Survey | General Population | Adult |  |
| 60 | CN | Reynolds L | Serve the people or close the sale? Profit-driven overuse of injections and infusions in China's market-based healthcare system | 8 | 2011 | Peer Reviewed Article | Urban & Rural | Guizhou | Assess the Irrational Use and Describe the Influential Factors | Cross-sectional and Qualitative | Population Based Survey; Interview | Clinician; Patient |  | Tertiary/Secondary; Primary |
| 61 | VN | Rheinländer T | Perspectives on child diarrhoea management and health service use among ethnic minority caregivers in Vietnam. | 7 | 2011 | Peer Reviewed Article | Rural | Lao Cai Province | Assess the Irrational Use and Describe the Influential Factors | Qualitative (in-depth interviews) | Population Based Survey; Interview | General Population | Children |  |
| 62 | CN | San N | Impact of national essential medicines system on antibiotics uses in primary medical institutions | 8 | 2011 | Peer Reviewed Article | Urban & Rural | 10 provinces of China | Assess the Irrational Use | Time Series or Surveillance | Facility Survey |  |  | Primary |
| 63 | CN | Song Y | Impact of the essential drug list on rational drug use in grassroots facilities | 8 | 2012 | Peer Reviewed Article | Urban & Rural | Anhui, Zhejiang, Ningxia and Shandong | Assess the Irrational Use | Time Series or Surveillance | Prescription Survey | Patient |  | Primary |
| 64 | CN | Sun Q | Analyzing the status of drug use in medical institutions at county, township and village level in Shandong and Ningxia | 9 | 2010 | Peer Reviewed Article | Rural | Shandong, Ningxia | Assess the Irrational Use and Describe the Influential Factors | Cross-sectional | Population Based Survey; Prescription Survey | Patient; General Population |  | Tertiary/Secondary; Primary |
| 65 | CN | Tang A | Analysis on current situation of drug application in township hospitals in rural poverty areas | 8 | 2002 | Peer Reviewed Article | Rural | Western China | Assess the Irrational Use | Cross-sectional | Prescription Survey | Patient |  | Primary |
| 66 | VN | Tran TT | Thực hành kê đơn phân phối thuốc tại phòng khám đa khoa hai bệnh viện Thiệu Hóa và Cẩm Thủy, tỉnh Thanh Hóa | 8 | 2012 | Grey Literature | Rural | 12 districts of Thanh Hoa province | Assess the Irrational Use | Time Series or Surveillance | Facility Survey; Prescription Survey; Medical record Review | Patient |  | Primary |
| 67 | VN | Tran TT | Thực trạng sử dụng thuốc thiết yếu qua hồi cứu đơn thuốc tại 12 xã thuộc hai huyện Thiệu Hóa và Cẩm Thủy, tỉnh Thanh Hóa | 8 | 2012 | Grey Literature | Rural | 12 districts of Thanh Hoa province | Assess the Irrational Use | Time Series or Surveillance | Facility Survey; Prescription Survey; Medical record Review | Patient |  | Primary |
| 68 | VN | Tran TT | Thực trạng tiếp cận thuốc tại12 xã thuộc hai huyện Thiệu Hóa và Cẩm Thủy, tỉnh Thanh Hóa | 7 | 2012 | Grey Literature | Rural | 12 districts of Thanh Hoa province | Assess the Irrational Use | Time Series or Surveillance | Facility Survey; Prescription Survey; Medical record Review | Patient |  | Primary |
| 69 | VN | Tran TT | Nghiên cứu thực trạng tiếp cận thuốc tại một số trạm y tế xã thuộc 24 tỉnh | 7 | 2012 | Grey literature | Rural | 24 province | Assess the Irrational Use | Cross-sectional | Facility Survey; Prescription Survey; Medical record Review | Health workders |  | Primary |
| 70 | VN | Trinh MH | Bước đầu khảo sát sự hiểu biết của người mua thuốc về các loại hình bán lẻ trên địa bàn thành phố Biên Hòa, tỉnh Đồng Nai | 7 | 2011 | Peer Reviewed Article | Urban | Bien Hoa City (Binh Duong province) | Assess the Irrational Use | Cross-sectional | Population Based Survey | General Population |  | Pharmacy |
| 71 | VN | Vu TTH | Đánhgiá hoạt động của hội đồng thuốc và điều trị trong xây dựng và thực hiện danh mục thuốc tại một số bệnh viện đa khoa | 8 | 2012 | PhD Dissertation | Urban & Rural | Nationwide | Assess the Irrational Use and Describe the Influential Factors | Cross-sectional & Qualitative | Facility Survey; Interview | Pharmacy Staff |  | Primary |
| 72 | CN | Wang J | Irrational drug use in 34 township hospitals of Dingxi during 2006-2008 | 9 | 2009 | Peer Reviewed Article | Rural | Gansu | Assess the Irrational Use | Time Series or Surveillance | Prescription Survey; Medical record Review | Patient |  | Primary |
| 73 | CN | Wang L | Impact of national essential medicines policies on rational medicines use in primary care | 9 | 2012 | PhD Dissertation | Urban & Rural | National Wide | Assess the Irrational Use and Describe the Influential Factors | Time Series or Surveillance | Population Based Survey; Prescription Survey | Patient |  | Primary |
| 74 | CN | Wang S | Status quo and countermeasures of national essential medicine system in township health centers of Zhejiang Province | 7 | 2011 | Peer Reviewed Article | Rural | Zhejiang | Assess the Irrational Use and Describe the Influential Factors | Time Series or Surveillance | Prescription Survey | Patient |  | Primary |
| 75 | CN | Wang X | Analysis of rational use of drug in community health service facilities in Xiamen City | 8 | 2009 | Peer Reviewed Article | Urban | Fujian | Assess the Irrational Use | Cross-sectional | Prescription Survey | Patient |  | Primary |
| 76 | CN | Wang Y | Investigation and survey of essential drugs provision and use in primary medical institutions from Guangdong Province | 8 | 2013 | Peer Reviewed Article | Urban & Rural | Guangdong Province | Assess the Irrational Use | Time Series or Surveillance | Prescription Survey | Patient |  | Primary |
| 77 | CN | Wang Z | Analysis of prescription drug use of village doctors in rural poverty areas in 9 provinces of the west China | 8 | 2003 | Peer Reviewed Article | Rural | 9 provinces in western china | Assess the Irrational Use | Cross-sectional | Prescription Survey | Patient |  | Primary |
| 78 | CN | Wu A | Study on the frequency of antibiotics use per day among inpatients in 151 hospitals in 2003 | 7 | 2005 | Peer Reviewed Article | Urban & Rural | country wide | Assess the Irrational Use and Describe the Influential Factors | Time Series or Surveillance | Medical record Review | Patient |  | Tertiary/Secondary |
| 79 | CN | Wu A | National healthcare-associated infection surveillance system point-prevalence survey of antimicrobial use in 740 Chinese hospitals in 2010 | 7 | 2012 | Peer Reviewed Article | Urban & Rural | National Wide | Assess the Irrational Use | Cross-sectional | Medical record Review | Patient |  | Tertiary/Secondary |
| 80 | CN | Xiao A | Investigation and analysis of drug use in the primary health institutions of Yunnan and Liaoning Province | 8 | 2011 | Peer Reviewed Article | Urban & Rural | Yunnan, Liaoning | Assess the Irrational Use | Cross-sectional | Prescription Survey | Patient |  | Primary |
| 81 | CN | Xie Z | Investigation and analysis on the antibacterial used in Meizhou countryside medical agencies | 9 | 2009 | PhD Dissertation | Rural | Guangdong | Assess the Irrational Use and Describe the Influential Factors | Time Series or Surveillance | Prescription Survey | Patient |  | Primary |
| 82 | CN | Yan J | Investigation and analysis on the development of special rectification campaign of clinical application of antibacterial in Ganzhou 27 second-class hospitals of Jiangxi | 8 | 2012 | Peer Reviewed Article | Urban & Rural | Jiangxi | Assess the Irrational Use | Cross-sectional | Prescription Survey; Medical record Review | Patient |  | Tertiary/Secondary |
| 83 | CN | Yang X | Research on rational drug use of prescription in 3-level medical organizations of western rural areas in China | 8 | 2006 | PhD Dissertation | Rural | Gansu, Ningxia and Qinghai | Assess the Irrational Use and Describe the Influential Factors | Time Series or Surveillance | Prescription Survey | Patient |  | Tertiary/Secondary; Primary |
| 84 | CN | Yin J | Study on drug use in rural area, Shandong Province and Ningxia Autonomous Region | 10 | 2009 | PhD Dissertation | Rural | Shandong, Ningxia | Assess the Irrational Use and Describe the Influential Factors | Cross-sectional | Population Based Survey; Prescription Survey | Patient; General Population |  | Tertiary/Secondary; Primary |
| 85 | CN | Yu L | Cross-sectional study on prescriptions in rural community health service stations of Shanghai | 8 | 2007 | Peer Reviewed Article | Rural | Shanghai | Assess the Irrational Use | Cross-sectional | Prescription Survey | Patient |  | Primary |
| 86 | CN | Yu Q | Analysis and effect study of national essential medicines system in primary medical institutions of Zhongshan Area | 8 | 2011 | Peer Reviewed Article | Urban | Guangdong | Assess the Irrational Use and Describe the Influential Factors | Time Series or Surveillance | Prescription Survey | Patient |  | Primary |
| 87 | CN | Zhang J | Cross-sectional survey on antimicrobial usage in patients at 108 hospitals in Guizhou province in 2010 | 7 | 2012 | Peer Reviewed Article | Urban & Rural | Guizhou | Assess the Irrational Use | Cross-sectional | Medical record Review | Patient |  | Tertiary/Secondary |
| 88 | CN | Zhang R | Utilization of antibacterial in 26 second-class and third-class hospitals in Shanghai during 2007-2009 | 7 | 2011 | Peer Reviewed Article | Urban | Shanghai | Assess the Irrational Use | Time Series or Surveillance | Facility Survey | Patient |  | Tertiary/Secondary |
| 89 | CN | Zhang W | Antibiotic use in five children's hospitals during 2002-2006: the impact of antibiotic guidelines issued by the Chinese Ministry of Health | 7 | 2008 | Peer Reviewed Article | Urban | Beijing, Shanghai, Guangzhou,Chongqing | Assess the Irrational Use and Describe the Influential Factors | Time Series or Surveillance | Facility Survey | Patient | Children | Tertiary/Secondary |
| 90 | CN | Zhang Y | Study on antibiotics use of lying-in women who attended the maternity insurance | 8 | 2012 | Peer Reviewed Article | Urban & Rural | Hunan | Assess the Irrational Use | Cross-sectional | Medical record Review | Patient | Adult |  |
| 91 | CN | Zhang Z | Adverse drug reaction and rational use of levofloxacin: a prospective study | 7 | 2001 | Peer Reviewed Article | Urban | Beijing | Assess the Irrational Use | Time Series or Surveillance | Medical record Review | Patient |  | Tertiary/Secondary |
| 92 | CN | Zhang Z | Research on implementation based on investigation of national essential drug system in Chenzhou | 5 | 2011 | PhD Dissertation | Urban | Hunan | Assess the Irrational Use and Describe the Influential Factors | Time Series or Surveillance | Population Based Survey; Prescription Survey | Clinician; Patient |  | Primary |
| 93 | CN | Zhou B | Effects of China's national essential medicines policy on the use of injection in primary health facilities | 8 | 2012 | Peer Reviewed Article | Urban & Rural | Liaoning，Shandong，Hubei，Shanxi，Sichuan and Shaanxi provinces | Assess the Irrational Use and Describe the Influential Factors | Time Series or Surveillance | Population Based Survey; Facility Survey;Prescription Survey | Patient |  | Primary |
| 94 | CN | Zhou B | An investigation of medicine use in rural hospitals and community health service centers in Chengdu | 7 | 2008 | Peer Reviewed Article | Urban & Rural | Sichuan | Assess the Irrational Use and Describe the Influential Factors | Cross-sectional | Facility Survey |  |  | Primary |
| 95 | CN | Zhou S | Investigation and analysis on the drug use in the out-patients prescriptions of primary medical and health institutions of Inner Mongolia in 2010 | 8 | 2012 | Peer Reviewed Article | Urban & Rural | Neimeigu | Assess the Irrational Use | Cross-sectional | Prescription Survey | Patient |  | Primary |
| 96 | CN | Zhou X | Analysis of quality on community health service in Nanchang City | 8 | 2009 | Peer Reviewed Article | Urban | Jiangxi | Assess the Irrational Use | Cross-sectional | Prescription Survey | Patient |  | Primary |

References:

1. Bai H (2008) Investigation of store state of family medicine-chest and drug taking safety. PhD Thesis. Central South University.
2. Bi P, Tong SL, Parton KA (2000) Family self-medication and antibiotics abuse for children and juveniles in a Chinese city. Soc Sci Med, 50(10):1445-1450.
3. Cai W, Lu Q, Zhang J, Feng J, Shi M, et al. (2006) Analysis of antibiotics uses in Shanghai during the first half of 2005. Chinese Journal of Pharmacoepidemiology, 15(4):227-230.
4. Chalker J, Chuc NT, Falkenberg T, Do NT, Tomson G (2000) STD management by private pharmacies in Hanoi: practice and knowledge of drug sellers. Sex Transm Infect, (4):299-302.
5. Chang X (2012) Research on the condition and influential factors of the prescribing in Beijing community health service centers. PhD dissertation. Peking Union Medical College.
6. Chen F, Bo Y, Xu Y, Ren H (2009) Retrospective analysis of compatible infusion prescriptions for outpatients and emergency patients of 11 hospitals in Tianjin. Chinese Journal of Hospital Pharmacy, (07):608-609.
7. Chen J, Fang W (2009) International indicators for rational drug use in outpatient prescriptions of 21 medical institutions in Shanghai Songjiang District. China Pharmacy, (19):1517-1518.
8. Chen W, Tang S, Sun J, Ross-Degnan D, Wagner AK (2010) Availability and use of essential medicines in China: manufacturing, supply, and prescribing in Shandong and Gansu provinces. BMC Health Serv Res, 10(1):211.
9. Chuc NT, Larsson M, Falkenberg T, Do NT, Binh NT, et al. (2001) Management of childhood acute respiratory infections at private pharmacies in Vietnam. Ann Pharmacother, 35(10):1283-1288.
10. Chuc NTK, Tomson G (1999) “Doi moi” and private pharmacies: a case study on dispensing and financial issues in Hanoi, Vietnam. Eur J Clin Pharmacol, 55(4):325-332.
11. Dong H, Bogg L, Rehnberg C, Diwan V (1999) Health financing policies: Providers' opinions and prescribing behavior in rural China. Int J Technol Assess. 15(4):686-698.
12. Dong L, Yan H, Wang D (2010) Polypharmacy and its correlates in village health clinics across 10 provinces of Western China. J Epidemiol Commun H, 64(6):549-553.
13. Dong L, Yan H, Wang D (2008) Antibiotic prescribing patterns in village health clinics across 10 provinces of Western China. J Antimicrob Chemoth, 62(2):410-415.
14. Dong L, Gao J, Yan H (2012) Comprehensive evaluation of prescription indicators for rational drug use of village doctors in 40 counties in west China. Chinese Journal of Health Statistics, 29(5):704-705.
15. Duong DV, Binns CW, Le TV (1997) Availability of antibiotics as over-the-counter drugs in pharmacies: a threat to public health in Vietnam. Trop Med Int Health, 2(12):1133-1139.
16. Han S, Liang H, Su W, Xue Y, Shi L (2013) Can Price controls reduce pharmaceutical expenses? A case study of antibacterial expenditures in 12 Chinese hospitals from 1996 to 2005. Int J Health Serv, 43(1):91-103.
17. He Z, Zhang D, Zhu M, Yuan W, Chen Y, et al. (2008) Evaluation of prescriptions in 12 secondary hospitals in Nantong area between June and Dec. in 2007. China Pharmacy, 19(29):2251-2254.
18. Hoa NQ, Larson, M, Chuc NTK, Erikson B, Trung NV, et al. (2009) Antibiotics and paediatric acute respiratory infections in rural Vietnam: health-care providers’ knowledge, practical competence and reported practice. Trop Med Int Health, 14(5):546-555.
19. Hoa NQ, Trung NV, Larsson M, Eriksson B, Phuc HD, et al. (2010). Decreased Streptococcus pneumoniae susceptibility to oral antibiotics among children in rural Vietnam: a community study. BMC Infect Dis, 10(1):85.
20. Hoa NQ, Öhman A, Lundborg CS, Chuc NTK (2007) Drug use and health-seeking behavior for childhood illness in Vietnam—a qualitative study. Health policy, 82(3):320-329.
21. Hoan LT, Chuc NTK, Ottosson E, Allebeck P. (2009) Drug use among children under 5 with respiratory illness and/or diarrhoea in a rural district of Vietnam. Pharmacoepidem Dr S, 18(6):448-453.
22. Hoang MH (2012) Hoạt động cung ứng thuốc tại bệnh viện Hữu Nghị – Thực trạng và một số giải pháp (Medicine supply in Huu Nghi Hospital – situation and solution) PhD Thesis. Hanoi Pharmacy School
23. Hoang TT, Nguyen TT, Duong HL, Tran TMO (2012) Thực trạng sử dụng thuốc cho nhóm bệnh nhân bảo hiểm y tế tại trạm y tế xã (The use of medicines for insured patients in community health clinics). Report for Health Policy and Strategy Institute.
24. Jiang M, Fang Y, Chen W, Yang S, Liu J, et al. (2013) Status quo on prescription antibiotics in retail pharmacies of Shaanxi Province. Chinese Journal of Health Policy, 6(1):40-45.
25. Jin C, Ely A, Fang L, Liang X. (2011) Framing a global health risk from the bottom-up: User perceptions and practices around antibiotics in four villages in China. Health Risk & Society, 13(5):433-449.
26. Kaljee LM, Anh DD, Minh TT, Tho LH, Batmunkh N, et al. (2011) Rural and urban Vietnamese mothers utilization of healthcare resources for children under 6 years with pneumonia and associated symptoms. Journal of Behavioral Medicine, 34(4): 254-267.
27. Larsson M, Krnovall G., Nguyen TKC, Karlsson I, Lager F, et al (2000) Antibiotic medication and bacteria resistance to antibiotics: a survey of children in a Vietnam community. Trop Med Int Health 5:711–721.
28. Larsson M, Tomson G, Binh NT, Chuc NTK, Falkenberg T (2006) Private pharmacy staff in Hanoi dispensing steroids-theory and practice. Pharmacy Practice, 4(2):60-67.
29. Larsson M, Falkenberg T, Dardashti A, Ekman T, Tornquist S, et al. (2005) Overprescribing of antibiotics to children in rural Vietnam. [Scand J Infect Dis.](http://www.ncbi.nlm.nih.gov/pubmed/16012004), 37(6-7):442-8.
30. Le TH, Ottosson E, Nguyen TKC, Kim BG, Allebeck P (2011) Drug use and self-medication among children with respiratory illness or diarrhea in a rural district in Vietnam: a qualitative study. Journal of Multidisciplinary Healthcare, 4:329.
31. Li F (2011) Availability of essential medicines research of community health services in Guangzhou. PhD Thesis. Guangzhou University of Chinese Medicine.
32. Li F, Xiong C, Cai J, Chang L, Wang X, et al. (2008) Investigation of utilization situation of antibiotics among residents in Beijing. Modern Preventive Medicine, 35(6):1199-1201.
33. Li J, Wang P, Wang X, Huang X (2003) Analysis on 82241 clinical prescriptions of 21 hospitals in Beijing. Clinical Medication Journal, 1(1):21-23.
34. Li L, Liu M (2012) Investigation of current situation of rational drug use among residents in three cities, Yunnan Province. Soft Science of Health, (9):819-822.
35. Li P (2009) Study on strategies of improving access to essential medicines in rural of China. PhD Thesis. Huazhong University of Science and Technology.
36. Li Y, Xu J, Wang F, Wang B, Liu L, et al. (2012) Overprescribing in China, driven by financial incentives, results in very high use of antibiotics, injections, and corticosteroids. Health Affairs, 31(5):1075-1082.
37. Li Y (2011) The status and effect study of the policy implementation of essential drug in Chinese city community health institutions. PhD Thesis. Huazhong University of Science and Technology.
38. Liang X, Jin C, Wang L, Wei L, Tomson G, et al. (2011) Unnecessary use of antibiotics for inpatient children with pneumonia in two counties of rural China. Int J Clin Pharm, 33(5):750-754.
39. Lin L (2012) Evaluation of the implementation of essential medicines system in Fujian primary care health institutions. PhD Thesis. Fujian Medical University.
40. Ling N, Hu C, Qiu YJ (2010) Analysis on current situation of intravenous infusion in the community health service stations of Zhongshan City. Chinese Community Doctors, 12(30):32-33.
41. Liu J (2011) Epidemiology study of prescription of antibacterial drugs of medical insurance inpatients in Changsha. PhD Thesis. Central South University.
42. Lu l, Liu Z, Zhang X (2009) Analysis of rational use of drug in community health service. Facilities. Chinese Health Economics, 28(4):45-47.
43. Luo H, Xiao S, Wang S (2012) Evaluation and analysis of outpatients and emergency prescriptions in 10 hospitals of Luzhou area and control measures. Herald of Medicine, 31(10):1387-1388.
44. Luo J (2009) Study on the status and influential factors of self-medication with antibacterial agents among customers in pharmacies. PhD Thesis. Central South University.
45. Luo J, Yan F, Wang W, Liu X, Lu C (2007) Medicine use and cost analysis by prescriptions of community health service institutions in Chengdu and Shenyang. Chinese Primary Health Care, 21(5):24-26.
46. Ma J, Ren R, Zhao J (2002) Medical costs and impacting factors on rural clinics of western rural areas in China. Chinese Primary Health Care, 16(8):1-4.
47. Mo R, Chen Y, Zhang X, Sun W (2007) Investigation and countermeasures study on irrational antibiotics use in the community health centers. Chinese General Practice, 14(25):2915-2917.
48. My HN, Gammeltoft T, Christoffersen SV, Thu TT, Rasch V (2009) Reproductive Tract Infections in Northern Vietnam: Health Providers' Diagnostic Dilemmas. Women & Health,49(2-3):229-245.
49. Nguyen TGH (2010). Nghiên cứu tính bất hợp lý trong chỉ định thuốc và đề xuất nâng cao tính hợp lý trong sử dụng thuốc tại một số bệnh viện tại miền Bắc, Việt Nam (Study on the irrational prescription of drugs and recommendations to promote the rational use of medicines in hospitals, Northern Vietnam). Report of a Ministry-level research project, Hanoi Medical School.
50. Nguyen TLH, Ho VH (2010). Nhiễm ký sinh trùng sốt rét và thực trạng sử dụng thuốc sốt rét tự điều tri của người dân ngủ rẫy tại Vĩnh Thạnh, tỉnh Bình Định (The prevalence of malaria parasite infection and usage of standby-treatment drugs among villagers who live and sleep in forest and farming areas). Journal of Military Medicines, 2010, No.4, Vol.35, p.13-16.
51. Nguyen H (2011) The principal-agent problems in health care: evidence from prescribing patterns of private providers in Vietnam. Health Policy Plann, 26(suppl 1), i53-i62.
52. O'Connor S, Rifkin D, Yang Y, Wang J, Levine OS, et al. (2001) Physician control of pediatric antimicrobial use in Beijing, China, and its rural environs. Pediatr Infect Dis J, 20(7):679-84.
53. Okumura J, Wakai S, Umenai T (2002) Drug utilisation and self-medication in rural communities in Vietnam. Soc Sci Med, 54(12): 1875-1886.
54. Pan H, Cui B, Zhang D, Farrar J, Law F, et al. (2012) Prior knowledge, older age, and higher allowance are risk factors for self-medication with antibiotics among university students in Southern China. Plos One, 7(7):e41314.
55. Phuong HL, De Vries PJ, Nagelkerke N, Giao PT, Hung LQ, et al. (2006) Acute undifferentiated fever in Binh Thuan province, Vietnam: imprecise clinical diagnosis and irrational pharmaco-therapy. Trop Med Int Health, 11(6):869-879.
56. Qin Y, Ren R (2006) The investigation of irrational use of medication in town hospitals and village health stations in Liaoning Province. Chinese Health Economics, 25(1):67-71.
57. Qu J, Meng Q, Zhang X, Li L, Li Yu, et al. (2006) An assessment on current situation of village clinics in Shandong Province--An assessment on standardization and quality of health services provided by village clinics. Chinese Health Economics, 25(2):29-31.
58. Quagliarello AB, Parry CM, Hien TT, Farrar JJ (2003) Factors associated with carriage of penicillin-resistant Streptococcus pneumoniae among Vietnamese children: a rural-urban divide. Journal of Health, Population and Nutrition, 316-324.
59. Ren Z, Yang W, Huang Y, Gu J, Fu Y, et al. (2012) A survey of knowledge and behavior on antibiotic use among young people in China. Progress in Modern Biomedicine, 12(15):2909-2913.
60. Reynolds L, McKee M (2011) Serve the people or close the sale? Profit-driven overuse of injections and infusions in China's market-based healthcare system. Int J Health Plann Manage, 26(4):449-70.
61. Rheinländer T, Samuelsen H, Dalsgaard A, Konradsen F (2011) Perspectives on child diarrhoea management and health service use among ethnic minority caregivers in Vietnam. BMC Public Health. 11(1):690.
62. San N, Fu H (2011) Impact of national essential medicines system on antibioics uses in primary medical institutions. Soft Science of Health, 25(11):782-785.
63. Song Y, Bian Y (2012) Impact of the essential drug list on rational drug use in grassroots facilities. Health Economics Research,(9):30-32.
64. Sun Q, Yan Y, Wang W, Bogg L, Tang S (2010) Analyzing the status of drug use in medical institutions at county, township and village level in Shandong and Ningxia. Chinese Health Service Management, 27(8):535-537,556.
65. Tang A, Yang H, Chen Y (2002) Analysis on current situation of drug application in township hospitals in rural poverty areas. Chinese Rural Health Service Administration, 22(10):59-60.
66. Tran TThoa, Truong Viet Dung, Nguyet Thi Thu, et al. (2012) Thực hành kê đơn phân phối thuốc tại phòng khám đa khoa hai bệnh viện Thiệu Hoa và Cẩm Thủy, tỉnh Thanh Hóa. Hanoi Medical School
67. Tran Thi Thoa, Truong Viet Dung, Nguyet Thi Thu, et al. (2012) Thực trạng sử dụng thuốc thiết yếu qua hồi cứu đơn thuốc tại 12 xã thuộc hai huyện Thiệu Hóa và Cẩm Thủy, tỉnh Thanh Hóa (Evaluation on the use of essential drugs by reviewing prescriptions from 12 commune clinics of Thieu Hoa and Cam Thuy districts, Thanh Hoa province). Hanoi Medical School
68. Tran TT, Truong VD, Pham QB, Nguyet TT (2012) Thực trạng tiếp cận thuốc tại12 xã thuộc hai huyện Thiệu Hóa và Cẩm Thủy, tỉnh Thanh Hóa. Hanoi Medical School.
69. Tran TT, Truong VD, Pham QB, Nguyet TT (2012) Nghiên cứu thực trạng tiếp cận thuốc tại một số trạm y tế xã thuộc 24 tỉnh (Study on accessibility of medicines and essential medicines at health care stations in 24 province). Hanoi Medical School.
70. Trinh MH (2011). Bước đầu khảo sát sự hiểu biết của người mua thuốc về các loại hình bán lẻ trên địa bàn thành phố Biên Hòa, tỉnh Đồng Nai (Informative survey on buyers’ knowledge about type of medicine retail stores in Bien Hoa City, Dong Nai Province). Journal of Military Medicines, 2011.
71. Vu TTH (2012). Đánhgiá hoạt động của hội đồng thuốc và điều trị trong xây dựng và thực hiện danh mục thuốc tại một số bệnh viện đa khoa (Assessment of the role and impact of committee and treatment in developing and implementing drug lists in general hospitals). PhD Thesis, Hanoi Pharmacy School.
72. Wang J, Guo Y, Yang S (2009) Irrational drug use in 34 township hospitals of Dingxi during 2006-2008. Evaluation and Analysis of Drug-Use in Hospitals of China, (11):861-863.
73. Wang L (2012) Impact of national essential medicines policies on rational medicines use in primary care. PhD Thesis. Fudan University.
74. Wang S, Huang X, Wang X (2011) Status quo and countermeasures of national essential medicine system in township health centers of Zhejiang Province. Chinese Journal of Health Policy, 04(6):30-34.
75. Wang X, Zhang Y (2009) Analysis of rational use of drug in community health service facilities in Xiamen City. China Journal of Pharmaceutical Economics,(4):17-21.
76. Wang Y, Wu H, Dang L (2013) Investigation and survey of essential drugs provision and use in primary medical institutions from Guangdong Province. China Pharmacy, (8):691-693.
77. Wang Z, Zheng Q, Yan H (2003) Analysis of prescription drug use of village doctors in rural poverty areas in 9 provinces of the west China. Chinese Rural Health Service Administration, 23(1):39-40.
78. Wu A, Ren N, Wen X, Xu X, Li J, et al. (2005) Study on the frequency of antibiotics use per day among inpatients in 151 hospitals in 2003. Chinese Journal of Epidemiology, 26(6):451-454.
79. Wu A, Li C, Wen X, Ren N (2012) National healthcare-associated infection surveillance system point-prevalence survey of antimicrobial use in 740 Chinese hospitals in 2010. Chinese Journal of Infection Control, 11(1):7-11.
80. Xiao A, Jin C, Fu W, Wei J, Jin D, et al. (2011) Investigation and analysis of drug use in the primary health institutions of Yunnan and Liaoning Province. China Pharmacy, (36):3449-3452.
81. Xie Z (2009). Investigation and analysis on the antibacterial used in Meizhou countryside medical agencies. M.Sc. Thesis. Jinan University.
82. Yan J, Guo C, Zhong B, Shi F (2012) Investigation and analysis on the development of special rectification campaign of clinical application of antibacterial in Ganzhou 27 second-class hospitals of Jiangxi. China Pharmacy, (16):1450-1452.
83. Yang X (2006). Research on rational drug use of prescription in 3-level medical organizations of western rural areas in China. PhD Thesis. Huazhong University of Science and Technology.
84. Yin J (2009) Study on drug use in rural area, Shandong Province and Ningxia Autonomous Region. M.Sc. Thesis. Shandong University.
85. Yu L, Zhang L, Zhang X, Wang D, Shen X (2007). Cross-sectional study on prescriptions in rural community health service stations of Shanghai. Chinese Rural Health Service Administration,27(7):512-514.
86. Yu Q, Liu Y, Li X, Deng B (2011) Analysis and effect study of national essential medicines system in primary medical institutions of Zhongshan Area. China Pharmaceuticals, 20(12):2-4.
87. Zhang J, Wang C, Xiong H, Yang H, Chen J, et al. (2012) Cross-sectional survey on antimicrobial usage in patients at 108 hospitals in Guizhou province in 2010. Chinese Journal of Infection Control, 11(6):430-434.
88. Zhang R, Ji Y, Jiang J, Mao S (2011) Utilization of antibacterial in 26 second-class and third-class hospitals in Shanghai during 2007-2009.Pharmaceutical Care and Research,11(4):258-261.
89. Zhang W, Shen X, Wang Y, Chen Y, Huang M, et al. (2008) Antibiotic use in five children's hospitals during 2002-2006: the impact of antibiotic guidelines issued by the Chinese Ministry of Health. Pharmacoepidem Dr S,17(3):306-311.
90. Zhang Y, He G, Liu J (2012) Study on antibiotics use of lying-in women who attended the maternity insurance. Chinese Journal of Health Statistics,29(2):247-248.
91. Zhang Z, Zhen J, Zhan S (2001) Adverse drug reaction and rational use of levofloxacin: a prospective study. Chinese Journal of Epidemiology, 22(6):405-407.
92. Zhang Z (2011) Research on implementation based on investigation of national essential drug system in Chenzhou. M.Sc. Thesis. University of South China.
93. Xiang X, Yang C, Wang D, Ye J, Zhang X. (2012). Effects of China’s national essential medicines policy on the use of injection in primary health facilities. J Huazhong U Sci-Med, 32:626-629.
94. Zhou B, Wan L, Li Y, Yu J, Yuan Z, et al. (2008) An investigation of medicine use in rural hospitals and community health service centers in Chengdu. West China University of Medical Science 8:529-537.
95. Zhou S (2012). Investigation and analysis on the drug use in the out-patients prescriptions of primary medical and health institutions of Inner Mongolia in 2010. China Pharmacy, (20):1839-1841.
96. Zhou X, Ruan S, Xi Q (2009) Analysis of quality on community health service in Nanchang City. Chinese Hospital Management, 29(1):61-63.
