# Supplementary material for: Systematic Review on Irrational Use of Medicines in China and Vietnam
Source: PLoS One. 2015 Mar 20;10(3):e0117710. doi: 10.1371/journal.pone.0117710 (PMC4368648; doi:10.1371/journal.pone.0117710)
Supplement: S2 Table — (DOCX) [file pone.0117710.s003.docx]

**Table S2 Sample size and key indicators of included studies**

Indicators were extracted directly from literatures and no extra calculation was conducted by reviewers.

|  | **Country** | **Tertiary or Secondary Hospital** | **Primary healthcare facility** | **Pharmacy** | **Outpatient** | **Inpatient** | **Prescription** | **Medical Record** | **Clinician** | **Household** | **Survey/questionnaire** | **# medication per prescription** | **% antibiotics** | **# antibiotics per encounter** | **% of injections** | **# injection per encounter** | **% of broad spectrum** | **% polypharmacy(>5)** | **% self-treat** | **% self-treat with antibiotics** | **% of wrong course** |
| --- | --- | --- | --- | --- | --- | --- | --- | --- | --- | --- | --- | --- | --- | --- | --- | --- | --- | --- | --- | --- | --- |
| 1 | CN | 0 | 0 | 0 | 0 | 0 | 0 | 0 | 0 | 211 | 0 |  |  |  |  |  |  |  |  |  |  |
| 2 | CN | 0 | 0 | 0 | 0 | 0 | 0 | 0 | 0 | 0 | 1459 |  | 41.8 |  |  |  |  |  | 59.4 |  |  |
| 3 | CN | 20 | 0 | 0 | 0 | 0 | 2000 | 540 | 0 | 0 | 0 |  | 70 |  |  |  |  |  |  |  |  |
| 4 | VN | 0 | 0 | 60 | 0 | 0 | 0 | 0 | 0 | 0 | 0 | 1.5 | 96.8 | 1.2 |  |  |  |  |  |  |  |
| 5 | CN | 0 | 312 | 0 | 0 | 0 | 37259 | 0 | 0 | 0 | 0 | 2.48 | 25 |  | 10.38 |  |  |  |  |  |  |
| 6 | CN | 11 | 0 | 0 | 0 | 0 | 8188 | 0 | 0 | 0 | 0 |  |  |  |  |  |  |  |  |  |  |
| 7 | CN | 7 | 14 | 0 | 0 | 0 | 16800 | 0 | 0 | 0 | 0 | 2.5 | 40 |  | 20.67 |  |  |  |  |  |  |
| 8 | CN | 0 | 63 | 122 | 0 | 0 | 5456 | 0 | 0 | 0 | 0 |  | 55.5 |  | 42.5 |  |  |  |  |  |  |
| 9 | VN | 0 | 0 | 0 | 0 | 0 | 0 | 0 | 0 | 0 | 0 |  | 35 |  |  |  |  |  |  |  | 64 |
| 10 | VN | 0 | 0 | 2 | 0 | 0 | 0 | 0 | 0 | 0 | 1833 |  | 22.6 | 2 |  |  | 90 |  |  | 94.9 | 95 |
| 11 | CN | 6 | 144 | 0 | 0 | 0 | 0 | 0 | 0 | 0 | 1064 |  |  |  |  |  |  |  |  |  |  |
| 12 | CN | 0 | 680 | 0 | 0 | 0 | 20125 | 0 | 680 | 0 | 0 | 2.36 |  |  | 50 |  |  | 5.8 |  |  |  |
| 13 | CN | 0 | 680 | 0 | 0 | 0 | 20125 | 0 | 0 | 0 | 0 |  | 48.43 |  |  |  |  |  |  |  |  |
| 14 | CN | 0 | 680 | 0 | 0 | 0 | 20125 | 0 | 0 | 0 | 0 |  | 48.43 |  | 22.93 |  |  | 15.91 |  |  |  |
| 15 | VN | 0 | 0 | 25 | 0 | 0 | 0 | 0 | 0 | 0 | 0 |  |  |  |  |  |  |  | 86.6 |  | 64.7 |
| 16 | CN | 12 | 0 | 0 | 0 | 0 | 0 | 0 | 0 | 0 | 0 |  |  |  |  |  |  |  |  |  |  |
| 17 | CN | 12 | 0 | 0 | 0 | 0 | 2520 | 0 | 0 | 0 | 0 | 2.1 | 37 |  | 21.2 |  |  |  |  |  |  |
| 18 | VN | 0 | 0 | 0 | 0 | 0 | 0 | 0 | 393 | 0 | 0 |  | 90 |  |  |  |  |  |  |  |  |
| 19 | VN | 0 | 0 | 0 | 0 | 0 | 0 | 0 | 0 | 0 | 818 |  | 58 |  |  |  | 49 |  |  |  | 42 |
| 20 | VN | 0 | 0 | 0 | 0 | 0 | 0 | 0 | 0 | 0 | 50 |  |  |  |  |  |  |  |  |  |  |
| 21 | VN | 0 | 0 | 0 | 0 | 0 | 0 | 0 | 0 | 0 | 4087 |  | 72.2 | 1.35 |  |  |  |  |  |  |  |
| 22 | VN | 1 | 0 | 0 | 0 | 0 | 0 | 0 | 0 | 0 | 0 |  |  |  |  |  |  |  |  |  |  |
| 23 | VN | 0 | 0 | 0 | 0 | 0 | 0 | 550 | 0 | 0 | 0 | 4.21; 3.65; 3.39 | 98.7 |  |  |  | 87.6 |  |  |  |  |
| 24 | CN | 0 | 0 | 213 | 0 | 0 | 0 | 0 | 0 | 0 | 426 |  |  |  |  |  |  |  |  |  |  |
| 25 | CN | 0 | 0 | 0 | 0 | 0 | 0 | 0 | 0 | 0 | 28 |  |  |  |  |  |  |  |  |  |  |
| 26 | VN | 0 | 0 | 0 | 0 | 0 | 0 | 0 | 0 | 362 | 0 |  |  |  |  |  |  |  | 12 |  | 40 |
| 27 | VN | 0 | 0 | 0 | 0 | 0 | 0 | 0 | 0 | 0 | 200 |  | 91 | 1.3 |  |  | 50 |  |  | 80 |  |
| 28 | VN | 0 | 0 | 0 | 0 | 0 | 0 | 0 | 70 | 0 |  |  |  |  |  |  | 76 |  |  |  |  |
| 29 | VN | 0 | 0 | 0 | 0 | 0 | 0 | 0 | 0 | 0 | 135 |  |  |  |  |  | 80 |  |  |  |  |
| 30 | VN | 0 | 0 | 0 | 0 | 0 | 0 | 0 | 5 | 0 | 30 |  | 100 |  |  |  | 20 |  | 88 |  |  |
| 31 | CN | 0 | 28 | 0 | 0 | 0 | 2800 | 0 | 0 | 0 | 0 |  | 47.16 |  | 37.32 |  |  | 3.03 |  |  |  |
| 32 | CN | 0 | 0 | 0 | 0 | 0 | 0 | 0 | 0 | 0 | 246 |  |  |  |  |  |  |  |  | 47 |  |
| 33 | CN | 7 | 14 | 0 | 0 | 0 | 82241 | 0 | 0 | 0 | 0 | 2.16 | 20.84 |  | 8.1 |  |  |  |  |  |  |
| 34 | CN | 0 | 0 | 0 | 0 | 0 | 0 | 0 | 0 | 0 | 1224 |  |  |  |  |  |  |  |  | 55.11 |  |
| 35 | CN | 6 | 12 | 0 | 0 | 0 | 540 | 0 | 0 | 0 | 0 | 2.5 |  |  | 50 |  |  |  |  |  |  |
| 36 | CN | 0 | 784 | 0 | 0 | 0 | 230800 | 0 | 0 | 0 | 0 | 2.6 | 50 |  | 33 |  |  |  |  |  |  |
| 37 | CN | 0 | 769.33 | 0 | 0 | 0 | 230800 | 0 | 0 | 0 | 0 | 2.51 | 43 |  | 34 |  |  |  |  |  |  |
| 38 | CN | 2 | 3 | 0 | 0 | 226 | 0 | 0 | 0 | 0 | 0 |  | 43.9 |  |  |  |  |  |  |  |  |
| 39 | CN | 0 | 17 | 0 | 0 | 0 | 7947 | 0 | 0 | 0 | 0 | 3.03 | 63.63 |  |  |  |  |  |  |  |  |
| 40 | CN | 0 | 27 | 0 | 0 | 0 | 4860 | 0 | 0 | 0 | 0 |  | 72.67 |  |  |  |  |  |  |  |  |
| 41 | CN | 0 | 0 | 0 | 0 | 0 | 0 | 245322 | 0 | 0 | 0 |  | 70.07 | 1.88 |  |  |  |  |  |  |  |
| 42 | CN | 0 | 744 | 0 | 0 | 0 | 74400 | 0 | 0 | 0 | 0 | 2.51 | 43.58 |  | 35.11 |  |  |  |  |  |  |
| 43 | CN | 10 | 0 | 0 | 0 | 0 | 6000 | 0 | 0 | 0 | 0 | 2.64 | 34.59 |  |  |  |  |  |  |  |  |
| 44 | CN | 0 | 0 | 112 | 0 | 0 | 0 | 0 | 0 | 0 | 1490 |  |  |  |  |  |  |  |  | 26.8 |  |
| 45 | CN | 0 | 15 | 0 | 0 | 0 | 2366 | 0 | 0 | 0 | 0 | 2.9 |  | 0.58 | 53 |  |  |  |  |  |  |
| 46 | CN | 0 | 450 | 0 | 0 | 0 | 13500 | 0 | 0 | 0 | 0 | 2.98 | 54.2 | 0.67 |  |  |  |  |  |  |  |
| 47 | CN | 0 | 32 | 0 | 0 | 0 | 3200 | 0 | 32 | 0 | 0 |  | 44.7 |  |  |  |  |  |  |  |  |
| 48 | VN | 0 | 0 | 0 | 0 | 0 | 0 | 0 | 10 | 0 | 748 |  |  |  |  |  |  |  |  |  |  |
| 49 | VN | 6 | 0 | 0 | 0 | 0 | 1359 | 885 | 0 | 0 | 0 | 4.7 | 60.6 |  |  |  | 15.1 ; 19 |  |  |  |  |
| 50 | VN | 0 | 0 | 0 | 0 | 0 | 0 | 0 | 0 | 0 | 227 |  |  |  |  |  |  |  |  |  | 71.05 |
| 51 | VN | 0 | 0 | 0 | 0 | 0 | 0 | 0 | 0 | 0 | 12300 | 3.85 |  |  | 34 |  |  |  |  |  |  |
| 52 | CN | 2 | 3 | 13 | 0 | 0 | 0 | 0 | 0 | 0 | 533 |  |  |  |  |  |  |  |  |  |  |
| 53 | VN | 0 | 0 | 0 | 0 | 0 | 0 | 0 | 0 | 0 | 505 |  |  |  |  |  |  |  | 55 |  |  |
| 54 | CN | 0 | 0 | 0 | 0 | 0 | 0 | 0 | 0 | 0 | 1300 |  |  |  |  |  |  |  |  | 47.8 |  |
| 55 | VN | 0 | 0 | 0 | 0 | 0 | 0 | 0 | 0 | 0 | 2096 |  |  |  |  |  |  |  | 65 |  | 45.6 |
| 56 | CN | 0 | 47 | 0 | 0 | 0 | 1461 | 0 | 0 | 0 | 0 | 2.56 | 60.8 | 0.73 | 43.9 | 0.175 |  |  |  |  |  |
| 57 | CN | 0 | 83 | 0 | 0 | 0 | 0 | 0 | 146 | 0 | 0 |  |  |  |  |  |  |  |  |  |  |
| 58 | VN | 0 | 0 | 75 | 0 | 0 | 0 | 0 | 0 | 0 | 529 |  | 99 |  |  |  | 84 |  | 42 |  |  |
| 59 | CN | 0 | 0 | 0 | 0 | 0 | 0 | 0 | 0 | 0 | 2630 |  |  |  |  |  |  |  |  | 77.32 | 52.09 |
| 60 | CN | 0 | 0 | 0 | 24 | 0 | 0 | 0 | 40 | 0 | 16 |  |  |  |  |  |  |  |  |  |  |
| 61 | VN | 0 | 0 | 0 | 0 | 0 | 0 | 0 | 0 | 0 | 43 |  |  |  |  |  |  |  |  |  |  |
| 62 | CN | 0 | 50 | 0 | 0 | 0 | 0 | 0 | 0 | 0 | 0 |  | 48.36 |  | 41.96 |  |  |  |  |  |  |
| 63 | CN | 0 | 151 | 0 | 0 | 0 | 26441 | 0 | 0 | 0 | 0 | 3.55 | 57.44 | 0.79 | 36.7 |  |  |  |  |  |  |
| 64 | CN | 3 | 12 | 0 | 0 | 0 | 3000 | 0 | 0 | 3228(13481) | 0 |  |  |  |  |  |  |  |  |  |  |
| 65 | CN | 0 | 230 | 0 | 0 | 0 | 6360 | 0 | 0 | 0 | 0 | 3.98 | 58.97 |  | 44.9 |  |  |  |  |  |  |
| 66 | VN | 0 | 2 | 0 | 0 | 0 | 0 | 0 | 0 | 0 | 0 | 3.7 | 91 |  |  |  | 53.7 |  |  |  |  |
| 67 | VN | 0 | 12 | 0 | 0 | 0 | 1440 | 0 | 0 | 0 | 0 |  |  |  |  |  |  |  |  |  |  |
| 68 | VN | 0 | 0 | 0 | 0 | 0 | 0 | 0 | 0 | 0 | 0 |  |  |  |  |  | 53 |  |  |  |  |
| 69 | VN | 0 | 176 | 0 | 0 | 0 | 0 | 0 | 0 | 0 | 0 |  | 19.6 |  |  | 19 |  |  |  |  |  |
| 70 | VN | 0 | 0 | 0 | 0 | 0 | 0 | 0 | 0 | 0 | 578 |  |  |  |  |  |  |  |  |  |  |
| 71 | VN | 21 | 0 | 0 | 0 | 0 | 0 | 0 | 0 | 0 | 0 |  |  |  |  |  | 67.4 |  |  |  |  |
| 72 | CN | 0 | 34 | 0 | 0 | 0 | 12240 | 2910 | 0 | 0 | 0 | 3.88 | 67.25 |  | 30.37 |  |  |  |  |  |  |
| 73 | CN | 0 | 83 | 0 | 784 | 0 | 8233 | 0 | 0 | 0 | 0 | 3.63 | 52.6 |  | 31.7 |  |  | 26.9 |  |  |  |
| 74 | CN | 0 | 15 | 0 | 0 | 0 | 0 | 1800 | 0 | 0 | 0 | 3.11 |  | 0.75 | 43 |  |  |  |  |  |  |
| 75 | CN | 0 | 20 | 0 | 0 | 0 | 2000 | 0 | 0 | 0 | 0 | 2.47 | 40.5 |  | 14.3 |  |  |  |  |  |  |
| 76 | CN | 0 | 54 | 0 | 0 | 0 | 3240 | 0 | 0 | 0 | 0 | 4.4 | 75 | 1.15 | 67 |  |  | 30 |  |  |  |
| 77 | CN | 0 | 464 | 0 | 0 | 0 | 13920 | 0 | 0 | 0 | 0 | 3.55 | 56.08 | 0.8 | 42.47 |  |  |  |  |  |  |
| 78 | CN | 151 | 0 | 0 | 0 |  | 89539 | 0 | 0 | 0 | 0 |  | 54.86 |  |  |  |  |  |  |  |  |
| 79 | CN | 740 | 0 | 0 | 0 | 407208 | 0 | 0 | 0 | 0 | 0 |  | 49.63 |  |  |  |  |  |  |  |  |
| 80 | CN | 0 | 20 | 0 | 0 | 0 | 22356 | 0 | 0 | 0 |  | 3.1 |  |  | 58.5 |  |  |  |  | 21.1 |  |
| 81 | CN | 0 | 30 | 0 | 0 | 0 | 16000 | 0 | 0 | 0 | 0 |  | 87.56 |  |  |  |  |  |  |  |  |
| 82 | CN | 27 | 0 | 0 | 0 | 0 | 2700 | 2700 | 0 | 0 | 0 |  |  |  | 48 |  |  |  |  |  |  |
| 83 | CN | 5 | 15 | 0 | 0 | 0 | 10812 | 0 | 0 | 0 | 0 | 3.13 | 45.3 |  | 26 |  |  | 24.7 |  |  |  |
| 84 | CN | 3 | 12 | 0 | 0 | 0 | 3000 | 0 | 0 | 3228(13481) | 0 | 3.31 | 66 |  | 48 |  |  | 12.43 | 35.7 |  | 74.3 |
| 85 | CN | 0 | 72 | 0 | 0 | 0 | 4896 | 0 | 0 | 0 | 0 | 1.93 | 37.9 |  | 12.3 |  |  |  |  |  |  |
| 86 | CN | 0 | 15 | 0 | 0 | 0 | 3000 | 0 | 0 | 0 | 0 | 4.6 | 65 |  | 58 |  |  |  |  |  |  |
| 87 | CN | 108 | 0 | 0 | 0 | 29358 | 0 | 0 | 0 | 0 | 0 |  | 55.52 |  |  |  |  |  |  |  |  |
| 88 | CN | 26 | 0 | 0 | 0 | 0 | 0 | 0 | 0 | 0 | 0 |  |  |  |  |  |  |  |  |  |  |
| 89 | CN | 5 | 0 | 0 | 0 | 0 | 0 | 0 | 0 | 0 | 0 |  | 65 |  |  |  |  |  |  |  |  |
| 90 | CN | 0 | 0 | 0 | 0 | 0 | 0 | 6834 | 0 | 0 | 0 |  |  |  |  |  |  |  |  |  |  |
| 91 | CN | 7 | 0 | 0 | 0 | 0 | 0 | 2661 | 0 | 0 | 0 |  |  |  |  |  |  |  |  |  | 80.6 |
| 92 | CN | 0 | 36 | 0 | 0 | 0 | 4800 | 0 | 367 | 0 | 0 | 3.4 | 55 |  | 41 |  |  |  |  |  |  |
| 93 | CN | 0 | 120 | 0 | 0 | 0 | 24000 | 0 | 0 | 0 | 0 |  |  |  | 38.9 |  |  |  |  |  |  |
| 94 | CN | 0 | 7 | 0 | 0 | 0 | 0 | 0 | 0 | 0 | 0 |  |  |  |  |  |  |  |  |  |  |
| 95 | CN | 0 | 58 | 0 | 0 | 0 | 5293 | 0 | 0 | 0 | 0 | 3.17 | 51.92 |  | 40.75 |  |  |  |  |  |  |
| 96 | CN | 0 | 20 | 0 | 0 | 0 | 2000 | 0 | 0 | 0 | 0 | 2.58 | 55.6 |  | 45.8 |  |  |  |  |  |  |
