# Supplementary material for: Systematic Review on Irrational Use of Medicines in China and Vietnam
Source: PLoS One. 2015 Mar 20;10(3):e0117710. doi: 10.1371/journal.pone.0117710 (PMC4368648; doi:10.1371/journal.pone.0117710)
Supplement: S3 Table — (DOCX) [file pone.0117710.s004.docx]

**Table S3 Manual searched list**

| Journal of Medical Research (VN) |
| --- |
| Journal of Military Medicine and Pharmacy (VN) |
| Journal of Practical Medicine (VN) |
| Journal of Vietnam Public Health |
| Institute of Health Strategy and Policy (VN) |
| Hanoi School of Medicines (VN) |
| Hanoi School of Pharmacy (VN) |
